# Numerical knockouts–In silico assessment of factors predisposing to thoracic aortic aneurysms

**DOI:** 10.1371/journal.pcbi.1008273

**Published:** 2020-10-20

**Authors:** M. Latorre, J. D. Humphrey

**Affiliations:** Department of Biomedical Engineering, Yale University, New Haven, CT, United States of America; Stanford University, UNITED STATES

## Abstract

Myriad risk factors–including uncontrolled hypertension, aging, and diverse genetic mutations–contribute to the development and enlargement of thoracic aortic aneurysms. Detailed analyses of clinical data and longitudinal studies of murine models continue to provide insight into the natural history of these potentially lethal conditions. Yet, because of the co-existence of multiple risk factors in most cases, it has been difficult to isolate individual effects of the many different factors or to understand how they act in combination. In this paper, we use a data-informed computational model of the initiation and progression of thoracic aortic aneurysms to contrast key predisposing risk factors both in isolation and in combination; these factors include localized losses of elastic fiber integrity, aberrant collagen remodeling, reduced smooth muscle contractility, and dysfunctional mechanosensing or mechanoregulation of extracellular matrix along with superimposed hypertension and aortic aging. In most cases, mild-to-severe localized losses in cellular function or matrix integrity give rise to varying degrees of local dilatations of the thoracic aorta, with enlargement typically exacerbated in cases wherein predisposing risk factors co-exist. The simulations suggest, for the first time, that effects of compromised smooth muscle contractility are more important in terms of dysfunctional mechanosensing and mechanoregulation of matrix than in vessel-level control of diameter and, furthermore, that dysfunctional mechanobiological control can yield lesions comparable to those in cases of compromised elastic fiber integrity. Particularly concerning, therefore, is that loss of constituents such as fibrillin-1, as in Marfan syndrome, can compromise both elastic fiber integrity and mechanosensing.

## Author summary

Aneurysms are local dilatations of the arterial wall that are responsible for significant disability and death. Detailed analyses of clinical data continue to provide insight into the natural history of these potentially lethal conditions, with myriad risk factors–including uncontrolled hypertension, aging, and diverse genetic mutations–contributing to their development and enlargement. Yet, because of the co-existence of these risk factors in most cases, it has been difficult to isolate individual effects or to understand how they act in combination. In this paper, we use a computational model of the initiation and progression of thoracic aortic aneurysms to contrast key predisposing factors both in isolation and in combination as well as with superimposed hypertension and aging. The present study recovers many findings from mouse models but with new and important observations that promise to guide in vivo and ex vivo studies as we seek to understand and eventually better treat these complex, multi-factorial lesions, with data-informed patient-specific computations eventually the way forward.

## Introduction

Thoracic aortic aneurysms (TAAs) are local dilatations of the proximal aorta that often have an increased risk of subsequent dissection and/or rupture. Clinical experience reveals that uncontrolled hypertension and natural aortic aging are key risk factors for the initiation and progression of these lesions [1,2], though many TAAs arise from diverse genetic mutations [3–5]. Specifically, predisposing mutations affect genes that encode extracellular matrix (e.g., *FBN1*, which encodes fibrillin-1, or *EFEMP2*, which encodes fibulin-4, both of which affect elastic fibers), cytoskeletal proteins (e.g., *ACTA2*, which encodes smooth muscle α-actin, and *MYH11*, which encodes smooth muscle myosin heavy chain), and the cytokine transforming growth factor-beta (*TGFB2*) or its signaling through transmembrane receptors (*TGFBR1*,*2*, which encode type 1 and 2 TGFβ receptors) or intracellular second messengers (e.g., *SMAD2*, which encodes a key downstream signaling molecule SMAD2), which affect collagen turnover among many other biological processes. There is, in addition, increasing evidence that detrimental alterations to either extracellular matrix or intracellular contractile proteins implicate a dysfunctional mechanotransduction axis in the smooth muscle cells as causative of certain TAAs [5–8].

Advances in genetics have provided critical insight into TAAs based on human data, though the complexity of the disease process continues to stimulate countless studies in mice, particularly those having mutations to genes that have been found in humans, including *Fbn1*, *Acta2*, *Myh11*, *Tgfbr1*, and so forth. Mouse models enable the collection of detailed longitudinal data on the molecular and cellular biology, histopathology, biomechanical properties, and in vivo cardiovascular function. Yet, these mouse models are similarly complex, with disease presentation differing with the wild-type background [9], manifesting at different times and to different extents within the same genetic model [10], and in some cases seemingly manifesting differently in mice and humans (cf. [11,12]). Indeed, genetic analyses reveal hundreds of differentially expressed genes even in the case of a targeted mutation to a single gene (e.g., [13,14]), suggesting that additional collateral or compensatory changes can complicate interpretation further.

There is, therefore, a pressing need to synthesize information from different types of studies, including cellular [15] and murine and clinical [16]. It has long been appreciated that the biomechanics is fundamental to understanding these complex lesions [17–19], with continuing suggestions that “biomechanical analyses should be an integral part of aortic disease characterization under different genetic lesions and pharmacological interventions” [2]. We submit, therefore, that data-informed computational models, including systems biological and biomechanical, can contribute to our understanding by synthesizing empirical information and generating and testing hypotheses (e.g., [20,21]). In this paper, we build on a well-established theoretical framework for describing arterial growth (changes in composition) and remodeling (changes in organization) that allows one to predict evolving geometry, microstructure, and mechanical properties. Specifically, we present computational simulations of the development of local dilatations of the murine thoracic aorta resulting from prescribed changes in many of the key risk factors that give rise to TAAs, including relative effects of local losses of elastic fiber integrity, aberrant collagen structure, reduced smooth muscle contractility, and dysfunctional mechanosensing or mechanoregulation of matrix, all of which have been implicated as causative in human and murine studies. In addition, we consider the effects of hypertension and aortic aging, both in isolation and when superimposed on local losses in matrix integrity or cell functionality. An advantage of in silico approaches is that one can study each of the implicated factors in isolation and then in combination. Results reveal that some local insults can have marked effects alone, but in most cases the combination of multiple insults or risk factors is particularly concerning.

## Methods

### Theoretical framework

The normal thoracic aorta consists of myriad constituents that collectively endow the wall with appropriate resilience and stiffness, which provide biomechanical functionality, and yet sufficient strength, which protects against mechanical failures such as dissection and rupture. Importantly, these constituents can turnover and allow the aorta to change its caliber, wall thickness, and composition, and thus biomechanical properties, to adapt to diverse changes in hemodynamic loading, as in consistent exercise or conversely hypertension [22,23]. In contrast, adverse changes occur in natural aging and most disease processes, which can result from phenotypic modulation of the cells (as in progressive endothelial dysfunction) and changes in matrix composition and organization (as in fibrosis). Hence, a computational modeling framework must account for different structurally significant constituents that can have different material properties and different rates of turnover.

Over a period of years, we developed a “constrained mixture model” that can describe the evolving composition, geometry, and nonlinear material properties of arteries under many conditions (e.g., [20,24,25]). Briefly, this model requires three different constitutive relations for each of the structurally significant constituents, which for convenience we lump into three groups: elastin-dominated matrix, collagen-dominated matrix, and functional smooth muscle cells. The three relationships describe the rates of mass density production (*m*^*α*^(*τ*)>0, where *τ* is the time at which constituent *α* = 1,2,…,*n* is produced), the removal of these constituents due both to their normal half-lives and accelerated losses in disease (*q*^*α*^(*s*,*τ*)∈[0,1], where *s* is the current time of interest), and the multiaxial mechanical properties (with passive behaviors captured by ${\hat{W}}^{\alpha }(F_{n(\tau )}^{\alpha }(s))>0$, which denotes the stored elastic energy in constituent *α* that depends on the multiaxial deformation $F_{n(\tau )}^{\alpha }(s)$ experienced by that constituent at current time *s* relative to the configuration at *τ* at which it was incorporated within the extant matrix) of the individual constituents. In particular, elastin is not produced in maturity, but it can become degraded or damaged; collagen turns over continuously, and functional smooth muscle (with passive and active contributions to load bearing) can increase (via cell proliferation), decrease (via apoptosis), or adapt (remodel).

Importantly, because of the mechanobiological control of matrix by intramural cells [26], the functions for rates of production and removal include (potentially evolving) nominal values ($m_{N}^{\alpha }(\tau )>0$ and $k_{N}^{\alpha }(\tau )>0$, respectively) as well as modulated changes that are proportional to deviations in mechanical stimuli from homeostatic values, here assumed in terms of a mean pressure- and axial force-induced intramural stress (*σ*) and flow-induced wall shear stress (*τ*_*w*_). Specifically, we model production (per unit current volume) by

$$m^{\alpha }(\tau )=m_{N}^{\alpha }(\tau )\Upsilon^{\alpha }(\tau )=m_{N}^{\alpha }(\tau )\left(1+K_{\sigma }^{\alpha }\Delta \sigma (\tau )-K_{\tau_{w}}^{\alpha }\Delta \tau_{w}(\tau )\right)$$ (1)

where $K_{i}^{\alpha }>0$ are gain-type parameters that dictate the sensitivity of the overall deviations in stress (Δ*σ* and Δ*τ*_*w*_) from homeostatic values (*σ*_*o*_ and *τ*_*wo*_), with Δ*σ* = ((1−*δ*)*σ*−*σ*_*o*_)/*σ*_*o*_ where *δ*∈[0,1] is a newly introduced parameter to account for perfect mechanosensing (*δ* = 0) or its dysfunction (*δ*≤1) and Δ*τ*_*w*_ = ((1−*ξ*)*τ*_*w*_−*τ*_*wo*_)/*τ*_*wo*_ where *ξ*∈[0,1] similarly accounts for endothelial health (*ξ* = 0) or its dysfunction (*ξ*≤1). These relations, generally collected into stimulus functions Υ^*α*^, capture, for example, increases in pressure-induced wall stress tending to heighten matrix production through increased angiotensin II and TGF*β* signaling as well as increases in flow-induced wall shear stress tending to attenuate matrix production via increased nitric oxide, which additionally stimulates vasodilatation [24,27]. In the simulations below, we assume that flow remains constant and that endothelial-mediated effects are confined to the non-aneurysmal segments; hence, we do not solve the full fluid-solid-growth problem here. We similarly assume that the distending pressure is constant, at either normotensive or hypertensive systolic levels, not considering pulse pressure due to the current lack of appropriate G&R relations in terms of pulsatility (cf. [28]).

We model removal by

$$q^{\alpha }(s,\tau )=\exp\left(-\int_{\tau }^{s}k_{N}^{\alpha }(t)dt\right)=\exp\left(-\int_{\tau }^{s}k_{o}^{\alpha }(1+k{\left(\Delta \sigma (t)\right)}^{2})dt\right)$$ (2)

where $k_{N}^{\alpha }$ are rate-type parameters modulating the role of deviations in intramural stress (Δ*σ*) from homeostatic values, with $k_{o}^{\alpha }$ the original rate, noting that increased wall stress can both increase the production / activation of proteinases and decrease the vulnerability of normal matrix to proteolytic digestion [29,30]. Production and removal of constituent *α* determine its homogenized mass density per unit reference volume through the convolution integral

$$\rho_{R}^{\alpha }(s)=\int_{-\infty }^{s}m_{R}^{\alpha }(\tau )q^{\alpha }(s,\tau )d\tau$$ (3)

where $m_{R}^{\alpha }=Jm^{\alpha }$ are associated referential mass density production rates, with $J=\sum \rho_{R}^{\alpha }/\rho =\det\;F$ the volume ratio computed from the deformation gradient **F** from reference to current (in vivo) configurations for the mixture.

Finally, the biomechanical behavior of the aortic wall is given in terms of the Cauchy stress

$$\sigma (s)=-p(s)I+\frac{2}{J(s)}F(s)\frac{\partial W_{R}(s)}{\partial C(s)}F^{T}(s)+\sigma^{act}(s)$$ (4)

where *p* is a Lagrange multiplier that enforces isochoric motions during transient loading, **C** = **F**^*T*^**F** the right Cauchy-Green tensor, $W_{R}=\sum W_{R}^{\alpha }$, that is, the total stored energy per unit reference volume *W*_*R*_ is the sum of the energies stored in the constituent parts (namely, with constituents *α* representing elastin- and collagen-dominated matrix as well as mechanically functional smooth muscle), and ***σ***^*act*^ is the contractile active stress generated by the smooth muscle cells. For the constituent-specific passive mechanical properties, we let

$$W_{R}^{e}(s)=\phi_{R}^{e}(s){\hat{W}}^{e}(s)=\phi_{R}^{e}(s)\frac{c^{e}}{2}(tr(F^{eT}(s)F^{e}(s))-3)$$ (5)

for the elastin-dominated matrix (*e*), where $\phi_{R}^{e}=J\phi^{e}$ represents its referential mass fraction within the mixture, **F**^*e*^ = **FG**^*e*^ is the deformation gradient measured from its fixed natural (stress-free) configuration, with **G**^*e*^ an effective deposition stretch tensor, and *c*^*e*^ is a shear modulus that can be determined from biomechanical testing data. For the energy stored in multiple fiber families, which turnover continuously, we let

$$W_{R}^{c,m}(s)=\frac{1}{\rho }\int_{-\infty }^{s}m_{R}^{c,m}(\tau )q^{c,m}(s,\tau ){\hat{W}}^{c,m}(\lambda_{n(\tau )}^{c,m}(s))d\tau$$ (6)

for collagen (*c*) and passive smooth muscle (*m*) dominated behaviors, where *ρ* is the mass density of the tissue, $m_{R}^{c,m}=Jm^{c,m}$ are referential mass density production rates, $\lambda_{n(\tau )}^{c,m}(s)$ the fiber stretches relative to the associated evolving natural configurations, including the extent of the respective deposition stretches *G*^*c*,*m*^ (which result directly from active cell mechanoregulation at the time of deposition), and

$${\hat{W}}^{c,m}\left(\lambda_{n(\tau )}^{c,m}(s)\right)=\frac{c_{1}^{c,m}}{{4c}_{2}^{c,m}}\left(\exp[c_{2}^{c,m}{\left({(\lambda }_{n(\tau )}^{c,m}{\left(s)\right)}^{2}-1\right)}^{2}]-1\right)$$ (7)

with $c_{j}^{i}$ material parameters that can be determined from biaxial mechanical tests on excised arteries. Moreover, we let [31]

$$\sigma^{act}(s)=\phi^{m}(s)T(\Delta \tau_{w}(s))\lambda_{\theta ,act}(s)\left(1-{\left(\frac{\lambda_{m}-\lambda_{\theta ,act}(s)}{\lambda_{m}-\lambda_{o}}\right)}^{2}\right)e_{\theta }⊗e_{\theta }$$ (8)

where *ϕ*^*m*^ is the mass fraction of smooth muscle, *T*(Δ*τ*_*w*_) = *T*_*max*_(1−exp(−(*C*_*B*_−*C*_*S*_Δ*τ*_*w*_)^2^)) is a flow induced wall shear stress mediated level of contractility of the circumferentially oriented smooth muscle cells (with *C*_*B*_ and *C*_*S*_ parameters), which exhibit a nonlinear dependence on the active circumferential stretch (*λ*_*θ*,*act*_), with *λ*_*m*_ and *λ*_*o*_ levels of stretch at which contraction is maximal or minimal. The active circumferential stretch *λ*_*θ*,*act*_(*s*) = *a*(*s*)/*a*_*act*_(*s*), with *a*(*s*) the current luminal radius and *a*_*act*_(*s*) an active reference length that describes adaptive shifts in vasomotor tone via rearrangement of smooth muscle cells via $a_{act}(s)=\int_{-\infty }^{s}k_{act}a(\bar{\tau })e^{-k_{act}(s-\bar{\tau })}d\bar{\tau }$ (cf. [27,31]) with *k*_*act*_ the associated rate parameter. In particular, *a*_*act*_(0) = *a*(0) (i.e., *λ*_*θ*,*act*_(0) = 1, given an in vivo reference configuration) and *a*_*act*_(*s*≫0)→*a*(*s*≫0) (i.e., *λ*_*θ*,*act*_(*s*≫0)→1) when active remodeling is complete.

### Model parameters

Recall that contributors to TAAs are thought to include compromised elastic fiber integrity and remodeled collagen, with altered turnover rates, dysfunctional or lost smooth muscle cells, and compromised mechanosensing and mechanoregulation of matrix in addition to the general risk factors of hypertension and aging. For purposes of illustration, we used biaxial biomechanical data from a normal descending thoracic aorta from a wild-type male mouse [25] to parameterize the baseline biomechanical model. Rather than determining the remaining parameters from specific mouse models of TAAs, to facilitate comparisons across the many different cases we varied parameters over reasonable ranges to model the different disease conditions of interest.

TAAs frequently present as asymmetric lesions and the involved segment need not be of uniform thickness or luminal diameter. Yet, to facilitate comparisons across cases and to increase insight into vessel-level consequences of the different insults, herein modeled constitutively, consider an initially straight cylindrical segment of the murine thoracic aorta of uniform in vivo wall thickness (*h*_*o*_ = 40 μm) and luminal radius (*a*_*o*_ = 647 μm) into which an initial insult can be placed within the central region, to avoid further complications due to end effects, either asymmetrically or axisymmetrically. Indeed, we focused primarily on axisymmetric cases, which enable the most consistent comparisons, with the evolving axisymmetric insult prescribed by

$$\vartheta (z_{o})=\vartheta_{end}+(\vartheta_{central}-\vartheta_{end})exp\left(-{\left|\frac{z_{o}-l_{o}/2}{z_{od}}\right|}^{\nu_{z}}\right)$$ (9)

where *z*_*o*_∈[0,*l*_*o*_], with *l*_*o*_ = 15 mm, is the axial coordinate in the reference (homeostatic) configuration, *ν*_*z*_ = 5 and *z*_*od*_ = 3 mm are respective (baseline) axial exponential decay and deviation parameters, and *ϑ*_*end*_ and *ϑ*_*central*_ are values of the associated controlling parameter near the ends (*z*_*o*_ = 0,*l*_*o*_) or within the central (*z*_*o*_ = *l*_*o*_/2) regions of the computational aorta. For example, the axial profile for $\vartheta =K_{\tau_{w}}^{\alpha }/K_{\sigma }^{\alpha }$, used in Eq (1) for all of the non-uniform simulations, is shown in Fig A in S1 Supporting Information. Values of key model parameters are listed in Table 1 (and associated caption) for both normal and diseased vessels. Based on preliminary finite element simulations, where different model parameters where altered gradually, we defined (Fig B in S1 Supporting Information), for the axisymmetric lesions, an ~30% loss of elastic fiber integrity as mild and an ~60% loss as severe, both captured by reductions in the value of the elastin-associated material parameter *c*^*e*^ (which is computationally equivalent to reducing the local mass fraction of elastin, see Eq (5)); this reduction of the value of *c*^*e*^ is comparable to that found via biaxial tests in the *Fbn1*^*mgR/mgR*^ model of Marfan syndrome [32]. Likewise, we defined mild-to-severe losses of collagen cross-linking by ~10 to 25% reductions in the material parameter $c_{1}^{c}$. Effects of increased collagen degradation relative to collagen production imply an increased rate *k*^*c*^; we used a decreased growth-feedback parameter $k^{c}K_{i}^{c}$ [33] or, equivalently, an increased ratio of the rate of smooth muscle-to-collagen turnover $\eta =(k^{m}K_{i}^{m})/(k^{c}K_{i}^{c})$ in our computations. Based on our preliminary study (e.g., Fig B in S1 Supporting Information), we defined mild-to-severe increases in collagen degradation relative to collagen production by 5 to 12.5% increments in the parameter *η*. We also defined mild-to-complete losses of contractile capacity by letting the value of the active stress parameter *T*_*max*_ be reduced by 60 to 100%. These preliminary simulations also revealed that the simulated growth and remodeling (G&R) responses were very sensitive to the parameter *δ*, which controls that percentage of the intramural stress that is mechano-sensed. Hence, we let mild-to-severe degrees of compromised mechanosensing be defined by *δ* = 0.075 to *δ* = 0.185. Compromised mechanoregulation of matrix was modeled by 0.4 to 1.2 percent reductions in the deposition stretch *G*^*c*^, another highly sensitive parameter, which relates to collagen undulation at the time of deposition.

**Table 1:** Representative values of parameters for the baseline model, a non-aneurysmal mouse descending thoracic aorta, adapted (homogenized through the thickness) from [25].

Inner radius, thickness, length	*a*_*o*_, *h*_*o*_, *l*_*o*_	0.647mm, 0.040mm, 15mm
Constituent mass fractions	$\phi_{o}^{e},\phi_{o}^{m},\phi_{o}^{c}$	0.34, 0.33, 0.33
Collagen relative fractions	*β*^*θ*^, *β*^*z*^, *β*^*d*^	0.056, 0.067,0.877
Diagonal collagen orientation	*α*_0o_	29.9°
Elastic material parameters	$c^{e},c_{1}^{m},c_{2}^{m},c_{1}^{c},c_{2}^{c}$	89.71kPa, 261.4kPa, 0.24, 234.9kPa, 4.08
Deposition stretches	$G_{\theta }^{e},G_{z}^{e},G_{r}^{e},G^{m},G^{c}$	$1.90,1.62,1/(G_{\theta }^{e}G_{z}^{e}),1.20,1.25$
Combined production-removal	$\eta =K_{i}^{m}/K_{i}^{c}∙k_{o}^{m}/k_{o}^{c}$	1
Shear-to-intramural gain ratio	$K_{\tau_{w}}^{m}/K_{\sigma }^{m}=K_{\tau_{w}}^{c}/K_{\sigma }^{c}$	0.35
Dysfunctional mechanosensing	*δ*,*ξ*	0,0
Vasoactive parameters	*T*_*max*_, *C*_*B*_, *C*_*S*_, *λ*_*m*_, *λ*_0_	250kPa, 0.8326, *C*_*B*_/2,1.1,0.4

**Fig 1:**
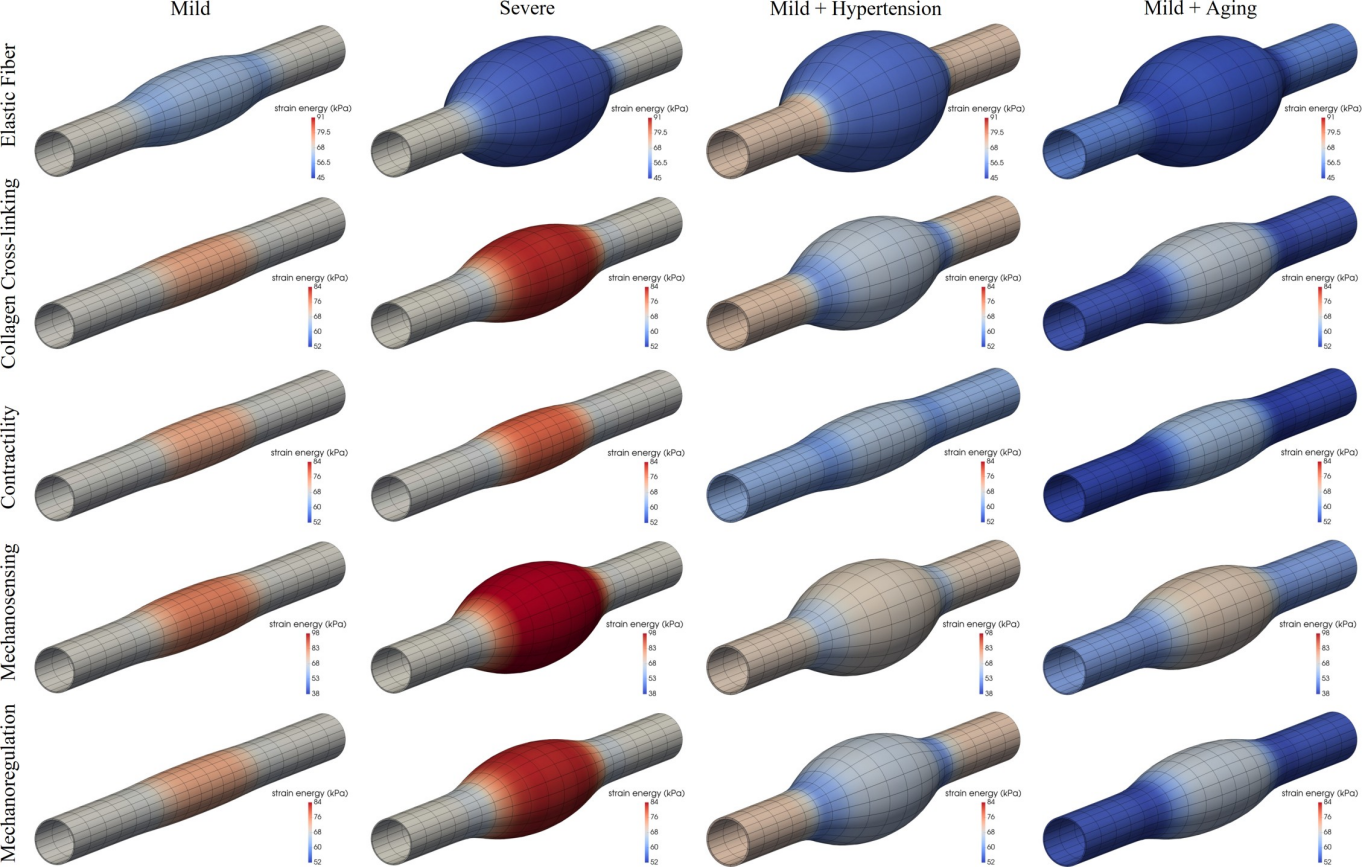
Fully developed, mechanobiologically equilibrated, axisymmetric dilatations of an initially cylindrical aortic segment caused by a mild (first column) or severe (second column) localized (via the respective parameter *ϑ*_*central*_ in Eq (9)) loss of elastic fiber integrity (first row, parameter *c*^*e*^, mild 32% and severe 62% reduction), decrease in collagen cross-linking (second row, parameter $c_{1}^{c}$, mild 9%, severe 23% reduction), loss of smooth muscle contractility (third row, parameter *T*_*max*_, mild 60%, severe 100% reduction), compromised mechanosensing (fourth row, parameter *δ*, mild 7.5%, severe 18.5%, absolute), or compromised mechanoregulation (fifth row, parameter *G*^*c*^, mild 0.4%, severe 1.2%, relative change), with the risk factors of hypertension (third column, 50% increase in blood pressure) and aortic aging (fourth column, uniform 30% loss of elastic fiber integrity) superimposed on the mild insults. Shown are color maps for elastic energy storage per unit current volume for the mechanically-loaded geometries. Note that the original in vivo homeostatic value *W*_*o*_ = 68 kPa (light grey, centered mark). Both ${K_{\tau_{w}}^{\alpha }/K_{\sigma }^{\alpha }|}_{end}>0$ and *η*_*end*_<1 were adjusted via Eq (9) to maintain the distal and proximal segments normal (cf. Figs A and F in S1 Supporting Information).

**Fig 2:**
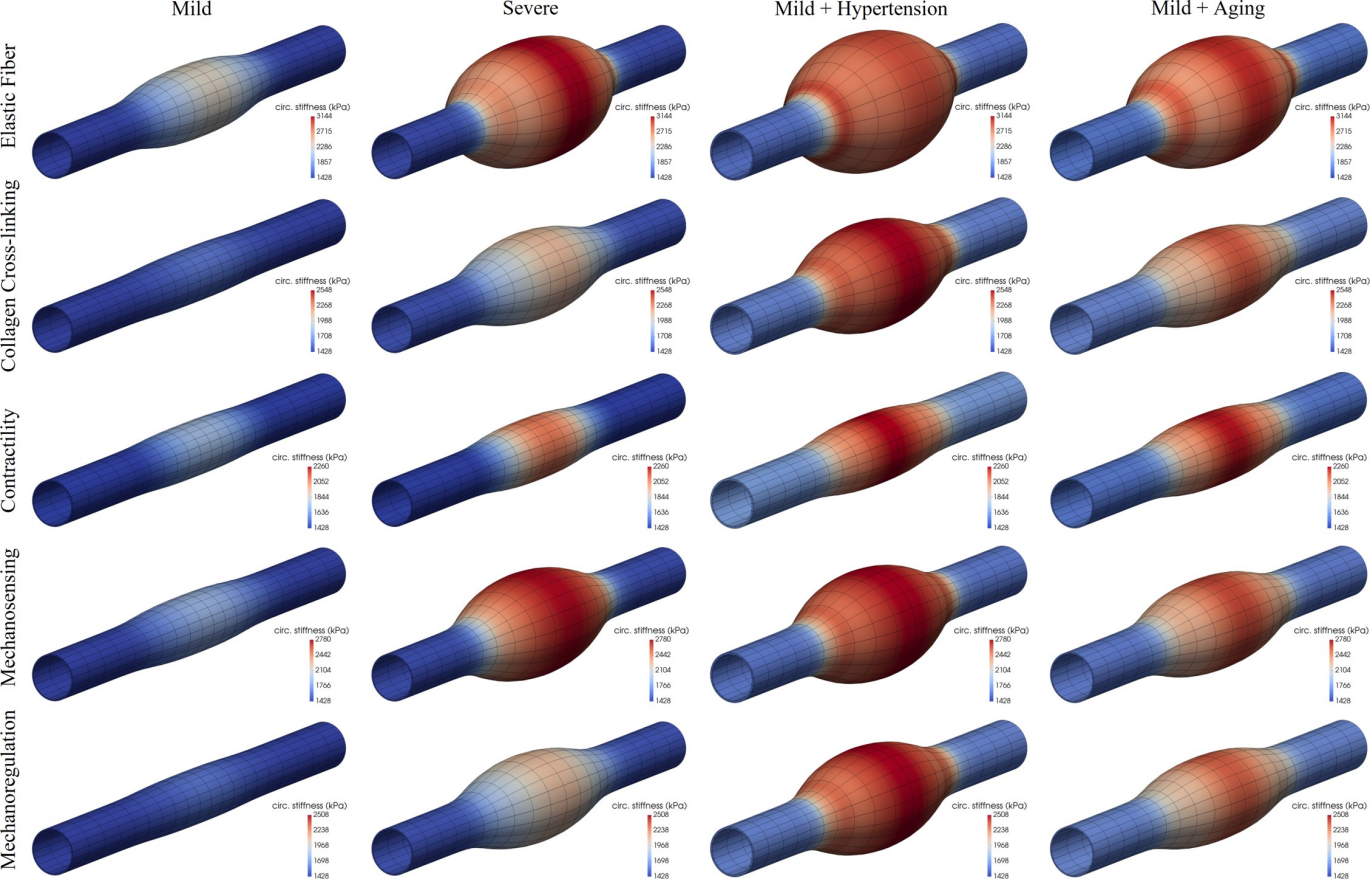
Same as Fig 1 except color maps for circumferential material stiffness for the mechanically-loaded geometries. Note the original in vivo homeostatic value *c*_*θθθθo*_ = 1428 kPa (dark blue, bottom mark) as well as an increased stiffness at the shoulder of some of the lesions resulting from a locally increased *α*_0*h*_ (i.e., higher *λ*_*θh*_/*λ*_*zh*_, see Eq (14)).

Superscripts *e*, *m*, *c* denote elastin-dominated, load-bearing smooth muscle, and collagen-dominated; superscripts/subscripts *r*, *θ*, *z*, *d* denote radial, circumferential, axial, and symmetric diagonal directions. Subscript *o* denotes original homeostatic values; subscripts *i* = *σ*, *τ*_*w*_ denote intramural and wall shear stress related parameters, respectively. For the severely diseased vessels, we let: $c_{central}^{e}=34.1\;kPa,\;c_{1|central}^{c}=181\;kPa,$ *T*_*max|central*_ = 0, *δ*_*central*_ = 0.185, $G_{central}^{c}=1.235$, or *η*_*central*_ = 1.05 for the axisymmetric aneurysms (see Eq (9) and Figs 1, 2, and H in S1 Supporting Information), and $c_{apex}^{e}=20.2\;kPa,\;c_{1|apex}^{c}=111\;kPa,$ *δ*_*apex*_ = 0.4, or $G_{apex}^{c}=1.22$ for the asymmetric ones (see Eq (10) and Fig 4), with *ϑ*_*end*_ = *ϑ*_*baseline*_ for all cases.

Finally, for completeness, we considered a few asymmetric simulations with the evolving asymmetric insult (i.e., axially- and circumferentially-nonuniform) prescribed by

$$\vartheta (z_{o},\theta_{o})=\vartheta_{end}+(\vartheta_{apex}-\vartheta_{end})exp\left(-{\left|\frac{z_{o}-l_{o}/2}{z_{od}}\right|}^{\nu_{z}}\right)exp\left(-{\left|\frac{\theta_{o}-\pi }{\theta_{od}}\right|}^{\nu_{\theta }}\right)$$ (10)

where *θ*_*o*_∈[0,2*π*] is the circumferential coordinate (azimuth) in the reference configuration, *ν*_*θ*_ = 5 and *θ*_*od*_ = *π*/3 are additional (baseline) circumferential exponential decay and deviation parameters, and *ϑ*_*apex*_ is the value of the associated controlling parameter at the apex (*z*_*o*_ = *l*_*o*_/2, *θ*_*o*_ = *π*) of the potentially developed aneurysm.

### Computational method

We have previously presented finite element implementations of our constrained mixture model (e.g., [34,35]), but herein we use a fast, efficient 3-D implementation [36] that is based on the underlying assumption that each G&R state is mechanobiologically equilibrated [27], which holds for cases wherein the characteristic timescale of the remodeling process is shorter than the timescale for the driving stimulus, that is, for fully quasi-static G&R [37,38].

Briefly, this rate-independent formulation eliminates dependence on G&R time *s* by letting responses “relax” towards evolved homeostatic states that satisfy mechanical and mechanobiological equilibrium at any time *s* (which, thus, plays the role of an evolution parameter). This allows equilibrium to be enforced in homeostatic grown and remodeled in vivo states for given external loads and boundary conditions without the need to track the past history of deposition and removal (as, e.g., in the heredity integral (Eq 6)) or to integrate evolution equations (as, e.g., in finite kinematic growth approaches [39]), hence resembling an elastic computation algorithmically. In particular, mechanobiological equilibrium in Eq (1) leads to (subscript *h* refers to evolved homeostatic variables)

$$\Upsilon_{h}^{\alpha }({\Delta \sigma }_{h},\Delta \tau_{wh})=1$$ (11)

whereby production balances removal (i.e., $m_{h}^{\alpha }=m_{Nh}^{\alpha }=k_{Nh}^{\alpha }\rho_{h}^{\alpha }$; [27]). On other hand, Eq (6) reduces to $W_{Rh}^{c,m}=\phi_{Rh}^{c,m}{\hat{W}}^{c,m}(G_{h}^{c,m})$, which, in combination with an equilibrated energy for elastin in Eq (5), leads to a rule-of-mixtures relation for the total energy $W_{Rh}=\sum \phi_{Rh}^{\alpha }{\hat{W}}^{\alpha }$ with evolved constituent mass fractions and deformations. Consistently, the Cauchy stress in Eq (4) also adopts a rule-of-mixtures expression in terms of evolved constituent-specific passive and active stresses

$$\sigma_{h}=\sum_{\alpha }^{e,c,m}\phi_{h}^{\alpha }{\hat{\sigma }}_{h}^{\alpha }+\phi_{h}^{m}{\hat{\sigma }}_{h}^{act}-p_{h}I$$ (12)

with the active contribution in Eq (8), and *λ*_*θ*,*act*,*h*_ = 1, relaxed as [27]

$$\sigma_{h}^{act}=\phi_{h}^{m}{\hat{\sigma }}_{h}^{act}(\Delta \tau_{wh})=\phi_{h}^{m}T(\Delta \tau_{wh})\left(1-{\left(\frac{\lambda_{m}-1}{\lambda_{m}-\lambda_{o}}\right)}^{2}\right)e_{\theta h}⊗e_{\theta h}$$ (13)

where both $\phi_{h}^{m}$ and Δ*τ*_*wh*_ are deformation-dependent, and the equilibrated (also deformation-dependent) Lagrange multiplier *p*_*h*_ is determined from the scalar stress-like constraint (Eq 11) during the quasi-static G&R evolution. An exact linearization of the formulation consistent with these constraints enables implementation within a finite element framework, where simultaneous solution of mechanical and mechanobiological equilibrium can be ensured efficiently at load steps that capture evolving geometries, compositions, and properties of interest for complex boundary value problems; see [36] for specific details.

Even if the present model considers each G&R state as mechanobiologically equilibrated, with degradation balanced by production, it still inherits useful ratios among relevant parameters from the full model, as, for example, those that control the rates of turnover of the individual constituents through the smooth muscle-to-collagen turnover ratio $\eta =\eta_{q}\eta_{\Upsilon }=(k^{m}/k^{c})(K_{i}^{m}/K_{i}^{c})$ mentioned above, whereby $\rho_{Rh}^{m}/\rho_{o}^{m}={\left(\rho_{Rh}^{c}/\rho_{o}^{c}\right)}^{\eta }$. Moreover, note that the deviation in shear stress from its homeostatic value Δ*τ*_*wh*_ = (1−*ξ*)*τ*_*wh*_/*τ*_*wo*_−1 in Eqs (11) and (13) must be assessed pointwise. We assume the analytical expression $\tau_{wh}/\tau_{wo}=Q_{h}a_{o}^{3}/(Q_{o}a_{h}^{3})$, with *a* luminal radius and *Q* blood flow rate, whose value and derivatives may be approximated and computed at Gauss points in terms of local circumferential and axial stretches *λ*_*θh*_ and *λ*_*zh*_ from reference to current homeostatic configurations (for cylindrical segments; see Appendix C in [36]), which, in turn, enables additional straightforward computations of Eq (13) and its consistent tangent from the equilibrated second Piola-Kirchhoff active stresses $S_{h}^{act}=J_{h}\phi_{h}^{m}U_{h}^{-1}{\hat{\sigma }}_{Nh}^{act}U_{h}^{-1}$ (cf. Eq (40) in [36]). For the intramural stress deviation in Eq (11), we consider $\sigma_{h}\equiv \sigma_{vh}=\frac{1}{3}tr(\sigma_{h})$ as a stimulus, which has repeatedly yielded numerical responses that reflect experimental observations well [25,36,40]. Moreover, for the sake of clarity in our prior presentation of this rate-independent computational framework [36], we did not posit specific evolution laws for the magnitude or orientation of deposition pre-stretches or -stresses; rather, the rotated (i.e., “right”) deposition stretch tensors $G_{Nh}^{c,m}$, and hence associated pre-stresses at the constituent level ${\hat{\sigma }}_{Nh}^{c,m}$, were prescribed during the evolution, which were particularly critical to control the anisotropic enlargement of the simulated aneurysms. Following previous studies on aneurysms (e.g., [34,35]), here we let the (referential) deposition angle *α*_0*h*_ of newly synthesized collagen fibers for the two symmetric diagonal families to evolve during G&R according to

$$\tan\alpha_{0h}=\frac{\lambda_{\theta h}}{\lambda_{zh}}\tan\alpha_{0o}$$ (14)

with *α*_0*o*_ given in Table 1. Rotated Cauchy pre-stress tensors ${\hat{\sigma }}_{Nh}^{c,m}(\alpha_{0h})$ for the diagonal fiber families reorient accordingly and require additional tangent contributions in terms of *α*_0*h*_(*λ*_*θh*_, *λ*_*zh*_) and its derivatives, see S1 Appendix. In this way, the present implementation extends the computational framework in [36] by considering an active stress for smooth muscle cells and reorientation of diagonal collagen fibers, both computed locally under mechanobiological equilibrium conditions.

Finally, many biomechanical metrics provide important insight into the state of aortic health or disease, including biaxial (circumferential and axial) wall stretch, stress, and material stiffness as well as elastic energy storage. Of these, elastic energy storage is a key indicator of mechanical functionality [41,42] while the value of circumferential material stiffness appears to be a particularly important indicator of aneurysmal propensity or presence [32,43]. Neither quantity can be measured directly; they are best computed from an appropriate nonlinear constitutive model of the aortic wall, modeled here with a 3-D finite element geometry. The finite element model for the initially straight cylindrical segment of the thoracic aorta comprised *N*_*r*_*N*_*θ*_*N*_*z*_ = 1×20×20 = 400 displacement-based 3-D quadratic elements with full 3×3×3 Gauss integration, which showed consistent results with other quadratic meshes analyzed (Fig C in S1 Supporting Information) as well as with a denser linear mesh in [36] while performing faster. Each finite element simulation was computed in a modified open source finite element code FEBio and advanced quasi-statically in 10 incremental steps, which showed quadratic rates of convergence during global Newton-Raphson iterations and elapsed total CPU times ~1 min on a single CPU processor at 3GHz in a Workstation Dell Precision 5810 with 32GB RAM. For all the simulations, axial displacements were fixed at the proximal and distal ends of the computational thoracic aorta, and cardiac output was considered constant (i.e., *Q*_*h*_/*Q*_*o*_ = 1).

## Results

### Hypertension and aging

Hypertension can arise via many causes and manifest as diverse vessel level changes, often associating with an increased structural stiffening of the central arteries, which in turn can feedback through the hemodynamics to increase pulse pressure further [44,45]. In particular, elevated blood pressure tends to stiffen the aorta structurally due primarily to an increased accumulation of fibrillar collagen [46]. Fig D in S1 Supporting Information shows simulated effects of a uniform elevation of systolic blood pressure of 1.5-fold on our baseline model of the murine thoracic aorta, with remodeling resulting in decreased elastic energy storage as well as a modestly increased circumferential material stiffness with an increased structural stiffness due to wall thickening. Vascular aging is similarly a complicated biological process, manifesting via many vessel level changes. In humans, a conspicuous change is diffuse elastic fiber fragmentation with an associated modest uniform dilatation and stiffening of the wall [47–49]. Fig D in S1 Supporting Information also shows simulated effects of an aging-related uniform loss of 30% of the elastic fibers relative to our baseline aortic model, which reflects well the general empirical observations, including a (irreversible) reduction in elastic energy storage [50]. Finally, the bottom panels in Fig D in S1 Supporting Information show aortic remodeling in response to a sustained elevation of pressure combined with such aging. Importantly, note that the aortic remodeling is diffuse, not localized, in all three of these cases. Effects of hypertension and aging on aortic remodeling are considered further below as possibly aggregating risk factors when combined with any of the five local factors that associate with TAAs and are contrasted herein.

### Elastic fiber integrity

Competent elastic fibers endow the aortic wall with an ability to store energy elastically during systole, which can then be used to work on the blood during diastole to augment flow, as, for example, to perfuse the coronary arteries of the left heart. Most histopathological studies reveal local fragmentation or loss of elastic fibers in human and murine TAAs (e.g., [14,51]). Figs 1 and 2 (first rows) show predicted results for locally compromised elastic fiber integrity both without and with the added risk factors of hypertension and aortic aging–these figures show the mechanically-loaded geometry after the lesion has developed, with colorimetric scaling for elastic energy storage (Fig 1) and circumferential material stiffness (Fig 2). As it can be seen, graded localized losses of elastic fiber integrity of ~30% and 60% can result in G&R responses that cause minor-to-marked localized dilatations of the aortic wall (cf. Fig B in S1 Supporting Information). Importantly, when either hypertension (a sustained increase in blood pressure, in this case a 50% elevation above baseline systolic) or aortic aging (arbitrarily taken as a uniform 30% loss in elastin) is superimposed on even the case of a mild localized loss of elastic fiber integrity, the otherwise unremarkable local dilatation increases dramatically. That is, hypertension and aortic aging can dramatically exacerbate the consequences of this underlying initiator of the localized dilatation. Note, too, that the maximal decrease in elastic energy storage capability and the maximal increase in circumferential material stiffness occur in the central, most dilated, region, with energy storage decreasing about 30% and stiffness increasing approximately 2-fold. These predicted changes are consistent with measured changes in the non-aneurysmal descending thoracic aorta in *Fbn1*^*mgR/mgR*^ mice, which have compromised elastic fiber integrity [32], though there was no attempt here to refine the model parameters to fit any particular data set. That the maximal changes co-localized with the region of maximal dilatation is also consistent with experimental (passive) measurements [43].

Finally, note that the loss of elastic fibers tends to associate with reduced vasoconstrictive capability [52], hence we did not initially include effects of smooth muscle contractility (i.e., in results displayed in Figs 1 and 2, first rows). Simulations in Fig E in S1 Supporting Information (left) show a potentially protective role of smooth muscle contraction (*T*_*max*_ = 50kPa; see Methods) when combined with a severe localized loss of elastic fiber integrity (~60%). Importantly, however, such an active resistance to dilatation (which would also be mediated by changes in the hemodynamics as the aorta enlarges) is limited, being overcome by an additional 10% localized degradation of elastin (right), thus leading to dilatations similar to those in Fig 1, although with further adverse changes in elastic energy storage and circumferential stiffness. Because all of these simulations depend on particular choices of multiple G&R model parameters, Fig F in S1 Supporting Information (first row) exams potential consequences of different values for the simpler case of a uniform mild (30%) loss of the elastic fiber integrity, which emphasizes further that (marked) aneurysmal dilatation results from (severe) localized, not uniform, losses in elastic fiber integrity, with various G&R factors simply modulating the specific extent of the response. Likewise, Fig G in S1 Supporting Information (first row) explores effects of size and spatial transition (from normal to diseased regions) of the axisymmetric lesion considered, which can be controlled with the decay (*ν*_*z*_) and deviation (*z*_*od*_) parameters in the Gaussian-like exponential distribution in Eq (9). Perhaps expectedly, for fixed minimal (*c*_*e*|*end*_) and maximal (*c*_*e*|*central*_) levels of compromised elastic fiber integrity, longer aneurysms develop for prescribed lesions that are initiated by greater axial insults (i.e., greater *z*_*od*_ for fixed *ν*_*z*_). Conversely, a notably reduced overall dilatation develops for a more gradual decay from maximally to minimally injured locations (i.e., lower *ν*_*z*_ for fixed *z*_*od*_). Similar conceptual predictions are obtained for the other insults considered below (second to fourth rows), although with different degrees of maximal aneurysmal dilatation.

### Collagen remodeling

Fibrillar collagens endow the aortic wall with stiffness and strength; they have a normal half-life on the order of months but can turnover more quickly to adapt the wall to changing mechanical loads. Such remodeling can also be maladaptive, however, resulting from or contributing to aneurysmal disease progression [53,54]. Collagen remodeling can manifest in many ways, including differences in cross-linking and rates of degradation. Figs 1 and 2 (second rows) show predicted effects of localized decreases in collagen cross-linking, both in isolation and in combination with hypertension or aging. As it can be seen, localized changes in collagen integrity also result in localized dilatations of the aortic wall, either modest or marked depending on the initiating insult, with all dilatations again exacerbated by hypertension or aging. Proteinase activity is increased in many TAAs [55], hence we similarly considered increased rates of collagen degradation relative to collagen production (via the parameter $\eta =(k^{m}K_{i}^{m})/(k^{c}K_{i}^{c})$; see Methods). Fig H in S1 Supporting Information shows predictions for a locally increased ratio *η* (Table 1) combined with either hypertension or aging, which again results in localized dilatations, particularly when both risk factors are superimposed. Importantly, complementing a prior finding that effects of flow-induced shear stress-mediated matrix turnover contribute to the biaxial G&R near the non-aneurysmal end regions [36], the parameter *η*>0 needed to be adjusted in the non-aneurysmal ends for all simulations to maintain the adjacent non-aneurysmal regions homeostatic (particularly those with uniform effects of aging). Indeed, a uniform moderate decrease of 20% in collagen cross-linking leads to minor-to-modest dilatations and stiffening depending on specific values of these G&R parameters, with a superimposed contractile capacity again proving to be potentially protective (Fig F in S1 Supporting Information, second row).

### Reduced smooth muscle contractility

Vascular caliber is controlled via multiple mechanisms, both global (central nervous system) and local, with the latter depending on endothelial cells sensing the local wall shear stress and secreting vasodilators or vasoconstrictors that induce smooth muscle relaxation or contraction [56,57]. Reduced smooth muscle contractility thus causes the wall to dilate, transferring load-bearing to the extracellular matrix [58]. Although elastic arteries such as the aorta are thought to have low basal tone compared with muscular arteries, the murine aorta has a significant contractile range. Figs 1 and 2 (third rows) show predicted consequences of mild and severe (total) localized losses of vessel-level smooth muscle tone in our model of the thoracic aorta. As it can be seen, overall effects on central geometry and material properties are relatively modest. Shown, too, is the predicted consequence of a localized loss of smooth muscle contractility with superimposed hypertension or aging. Perhaps surprisingly, overall effects on the geometry and properties are again modest. Albeit not shown, only when both hypertension and aging were superimposed on the case of a mild localized loss of smooth muscle contractility did the aortic dilatation and associated changes in biomechanical properties become dramatic. Aging is often accompanied by hypertension, hence emphasizing the need to consider many factors in combination. Finally, Fig F in S1 Supporting Information (third column) shows different responses given a uniform loss of 80% of smooth muscle tone combined with different G&R parameters, which again yields the most modest changes in geometry and stiffness among all of the considered insults.

### Dysfunctional mechanosensing

**Fig 3:**
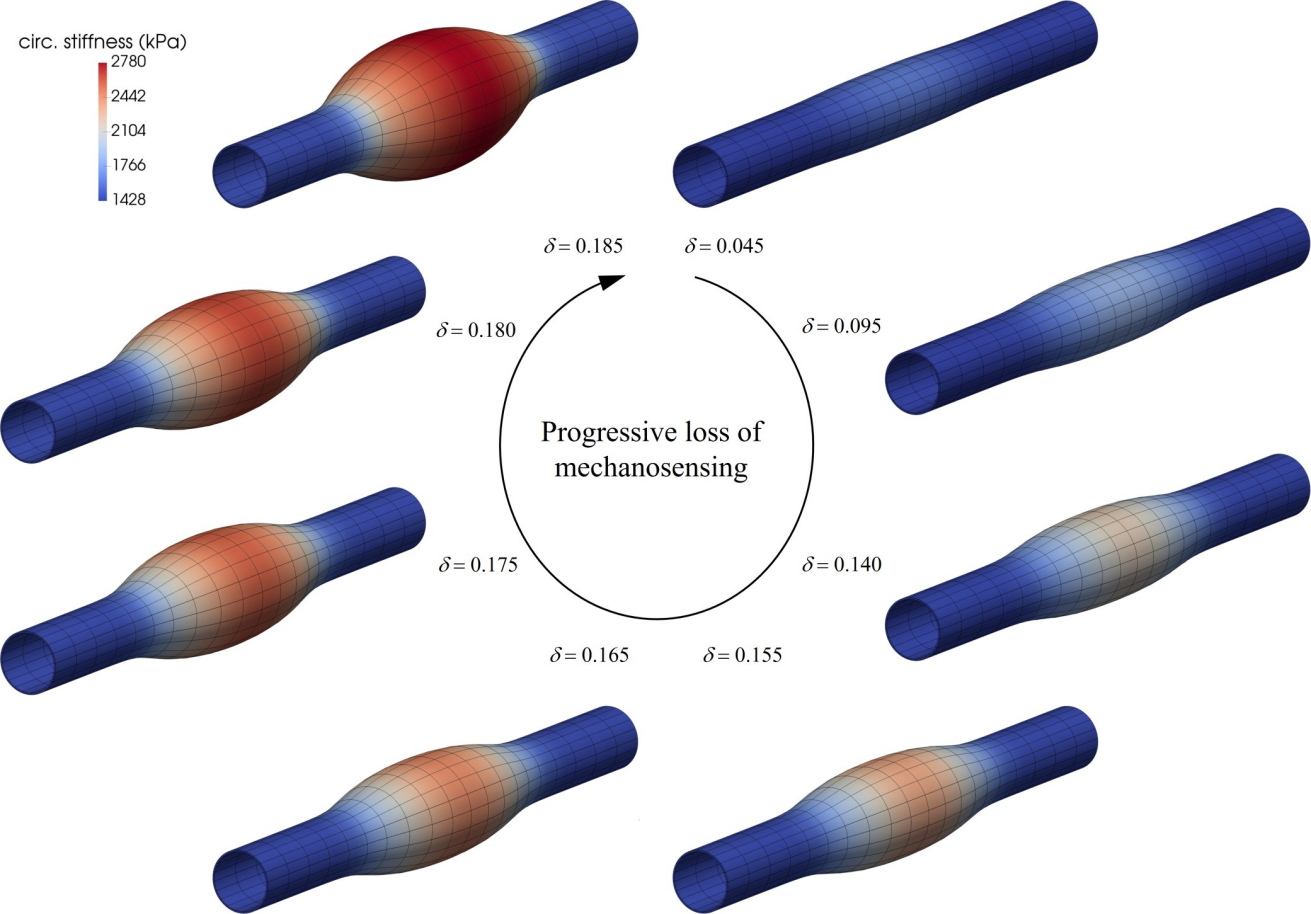
Mechanobiologically (quasi)equilibrated, progressive dilatation of an initial non-aneurysmal aortic segment with increasingly greater losses of mechanosensing *δ*>0 (clockwise from the upper right mesh). Note that the dilatation tended to asymptotic for *δ*→0.185^+^, suggesting the presence of a mechanobiological static instability [33] while maintaining inner pressure *P*_*h*_ = *P*_*o*_. Indeed, equilibrium solutions for *δ*≥0.185^+^ could not be achieved using standard Newton-Raphson global finite element iterations (hence defining the severe case in Figs 1 and 2 at *δ* = 0.185), suggesting the need for path-following (e.g., arc-length) methods to better capture numerically, and advance quasi-statically, these nearly-singular unstable responses.

Intramural cells sense their local mechanical environment, via integrins and competent actomyosin activity, by probing the extracellular matrix that carries most of the hemodynamically imposed load [59,60]. Such mechanosensing of matrix is thus critical to mechanical homeostasis of the aorta. Figs 1 and 2 (fourth rows) show predictions for mild and modest degrees of compromised mechanosensing, which importantly results in localized dilatations of the aortic wall that are exacerbated by hypertension and, to a lesser degree, aortic aging. Again, note that the maximal changes in elastic energy storage and circumferential material stiffness manifest within the most dilated region, with quantitative changes similar to those for compromised elastic fiber integrity (cf. first rows). Fig I in S1 Supporting Information shows predicted changes in additional biomechanical metrics–biaxial wall stress and material stiffness as well as local changes in volume ratio–due to the associated localized dilatations, again revealing marked changes. Fig 3 further shows a possible progressive (quasi-equilibrated mechanobiologically) dilatation with increasingly greater losses of mechanosensing. Finally, Fig F in S1 Supporting Information (fourth row) shows different responses given a uniform moderate (12%) loss of mechanosensing combined with different G&R and contractile parameters, with minor-to-modest dilatations and stiffening similar to predictions for a mild loss of elastic fiber integrity (first row).

### Dysfunctional mechanoregulation

Functional intramural cells not only degrade old structural constituents and deposit new constituents within the extant matrix, they work on and fashion these new constituents so as to ensure preferred properties under conditions of interest [61,62]. Mechanoregulation of collagen can be captured, in part, via values of the deposition stretches (or, pre-stresses) that are built into the new constituents at the time of matrix production. Figs 1 and 2 (fifth rows) show predicted effects of localized changes in the deposition stretch of the multiple families (i.e., orientations) of fibrillar collagen. Clearly, poorly mechanoregulated collagen within the central region can result in modest-to-marked localized dilatations of the aortic wall, which are again exacerbated by hypertension or aging. Consistent with the stress derived from Eq (7), decreased collagen cross-linking and dysfunctional mechanoregulation, as modeled here, affect the mechanical response of collagen similarly, hence leading to similar computational results when prescribed locally (cf. second rows), but also uniformly (Fig F in S1 Supporting Information, second and fifth rows). Note that it has been shown previously that values of the deposition stretch must be greater than unity to enable potentially optimal tissue maintenance or remodeling [24,63].

### Evolving stress-stretch relations

Biomechanical metrics such as material stiffness, energy storage, and contractility are fundamental descriptors of aortic properties and function [41,64], yet many people intuit changes in stress and stretch more easily. Hence, equibiaxial stress-stretch tests at the apices of the respective aneurysms were simulated, with material properties and mass fractions extracted in post-processing from our 3-D computational formulation. Fig J in S1 Supporting Information shows (passive) material behaviors after the respective lesions have arisen from marked losses of elastin fiber integrity (recall Figs 1 and 2, first rows), reductions in collagen cross-linking (second rows), decreased vessel-level smooth muscle contractility (third rows), dysfunctional mechanosensing (fourth rows), and dysfunctional mechanoregulation (fifth rows). Comparisons with the initial non-aneurysmal behaviors show a general loss of distensibility and extensibility in all five cases, consistent with the computed increase in material stiffness, even for those insults that do not alter directly the initial (passive) material properties, but rather the mass fractions (such as loss of contractility and dysfunctional mechanosensing). Note that the largest change in material behavior occurs in the circumferential and axial directions for the case of compromised elastic fiber integrity, which shows an initially compliant behavior that stiffens within the region of physiological stresses (~200 kPa). Consistent with the growth and remodeling observed in Figs 1 and 2, the smallest change in passive material behavior occurred for the loss of vessel-level smooth muscle contractility. Effects of reduced collagen cross-linking and compromised mechanosensing and mechanoregulation of matrix yield stiffer responses, although with differently evolved anisotropy. Importantly, the present results, which focus on differences in remodeled properties for aneurysms that develop from a common non-aneurysmal aorta, complement but extend significantly those in [35], which revealed a clear dependency of aneurysmal enlargement on initially different non-aneurysmal aortic properties, hence highlighting the complexity of the disease process.

### Asymmetric dilatations

**Fig 4:**
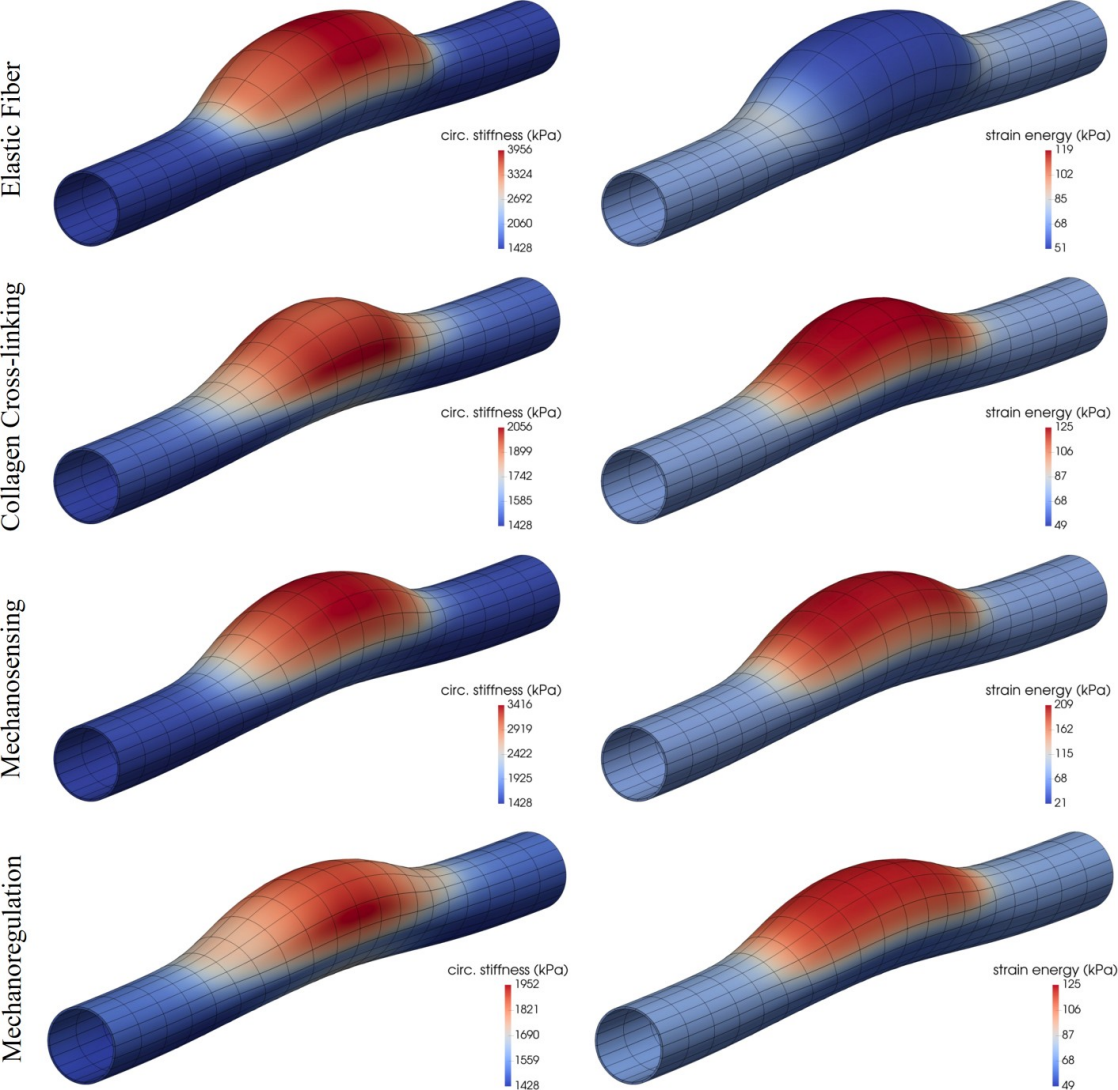
Fully developed, mechanobiologically equilibrated, asymmetric dilatations of an initially cylindrical aortic segment caused by marked locally compromised elastic fiber integrity (first row, 78% decrease in *c*^*e*^), collagen cross-linking (second row, 53% decrease in $c_{1}^{c}$), mechanosensing (third row, *δ* = 0.4), or mechanoregulation (fourth row, 2.3% decrease in *G*^*c*^) via *ϑ*_*apex*_ in Eq (10). Shown are color maps for circumferential material stiffness (left) and elastic energy storage per unit current volume (right) for the mechanically-loaded geometries. Note the original in vivo homeostatic values *c*_*θθθθo*_ = 1428 kPa (dark blue, bottom mark) and *W*_*o*_ = 68 kPa (light blue, second-from-bottom mark). Both ${K_{\tau_{w}}^{\alpha }/K_{\sigma }^{\alpha }|}_{end}>0$ and *η*_*end*_<1 were adjusted via Eq (9) to maintain the distal and proximal segments normal (cf. Figs A and F in S1 Supporting Information).

Although axisymmetric simulations are both computationally less expensive (e.g., by allowing one to focus on a single longitudinal section) and easier to interpret, most TAAs are asymmetric. Hence, we considered one class of asymmetric losses of cell or matrix function (Fig 4). Interestingly, findings were similar to those for the axisymmetric case although the degree of the more localized insult needed to be greater to elicit similar localized dilatations. In other words, cases of complete circumferential involvement of the initiator of localized dilatation can give rise to marked dilatations even if the degree of the insult is mild. When truly localized, axially and circumferentially, the surrounding normal tissue appears to lessen or slow the severity of the response. Indeed, Fig K in S1 Supporting Information shows effects of the length and width of the initiating axisymmetric insults considered, controlled in these cases with deviation parameters *z*_*od*_ and *θ*_*od*_ in the bidirectional Gaussian-like exponential distribution in Eq (10). Similar to the analysis in Fig G in S1 Supporting Information, for fixed minimal (*ϑ*_*end*_) and maximal (*ϑ*_*apex*_) levels of compromised parameters, longer and/or wider asymmetric aneurysms develop for prescribed insults that expand axially and/or circumferentially (i.e., greater *z*_*od*_ and/or *θ*_*od*_ for fixed *ν*_*z*_ and *ν*_*θ*_), with damage increased along the cross-sections generally leading to a substantial worsening of the aneurysms. Albeit not shown, the computational model predicted that diagonal fibers of collagen within the damaged area of these asymmetric aneurysms reoriented toward the circumferential direction, via Eq (14), consistent with a prior parametric analysis [36] that showed that asymmetric axial expansions of the central region, hence larger out-of-plane deformations of the aortic centerline associated with increasing realignments toward the circumferential direction (as prescribed therein).

## Discussion

Uncontrolled hypertension is a well-known risk factor for thoracic aortopathies and anti-hypertensive medications are often prescribed [2]. Hypertension increases the pressure-load on the aortic wall, which increases wall stress and the potential for mechanical failure, namely dissection or rupture. In addition, however, increased pressure-loading elicits mechanosensitive responses in normal cells that initially attempt to adapt the wall to offset the increased load, typically via the deposition of additional fibrillar collagen among other changes in composition. This hypertensive response is complex, however, often accompanied by endothelial dysfunction, smooth muscle cell phenotypic modulation, and inflammatory cell infiltration as well as global effects on the central nervous and renin-angiotensin systems [44,65]. Our model of hypertension focused on the initial mechano-adaptive response that seeks to return wall stress toward normal via an increased deposition mainly of collagen, which thickens the wall [25]. Vascular aging is similarly a well-known risk factor for many cardiovascular diseases, including TAAs [1]. Again, the effects of aging on the vasculature are manifold, including endothelial dysfunction, smooth muscle phenotypic modulation or apoptosis, extracellular matrix remodeling, and inflammation [66,67]. Our model of aging focused on the diffuse loss of elastic fiber integrity, as in human though not natural murine aging [50]. Consistent with observations in clinical and animal model studies, our in silico simulations yielded an aortic wall that remodeled in hypertension primarily via an increase in wall thickness and in aging primarily via an increase in caliber, both uniformly and both consistent with an initial mechano-adaptative response (i.e., in the absence of intramural cell dysfunction or inflammation). There were two key observations. First, neither hypertension nor aging alone resulted in localized dilatations; rather their effects were uniform in our idealized model. Second, both of these factors exacerbated each of the five different initiators of localized dilatation, which in many cases (with ≈170% and ≈100% maximal increases in diameter locally for axisymmetric and asymmetric lesions, respectively) reached the clinical definition of an aneurysm (≥50% increase). Our findings are thus consistent with these two conditions being important risk factors for aneurysmal progression, though not aneurysmal initiation consistent with clinical experience of millions of individuals who are hypertensive or of advanced age and yet the much smaller numbers that present with TAAs.

Competent elastic fibers endow the aortic wall with an ability to resist, indeed to recoil against, distending pressures, which helps to control luminal diameter. We considered localized losses of elastic fiber integrity as arise in Marfan syndrome. Studies in the *Fbn1*^*mgR/mgR*^ mouse model of Marfan syndrome reveal compromised elastic fiber integrity along the entirety of the aorta, though the greatest losses manifest in the aortic root/ascending aorta where aneurysms first develop [68]. It has been suggested that the different embryonic lineage of smooth muscle cells in this region (from the second heart field and neural crest) and the different state of mechanical loading (biaxial loading and complex hemodynamics) contribute to the predisposition of the proximal aorta to aneurysm [69–71]. We did not attempt to model propensity due to differential embryonic lineage or site-specific loading. Rather, we focused on the more general question as to whether a localized loss of elastic fiber integrity can result in aneurysmal dilatation based solely on a mechanobiological response to the insult. Consistent with prior simulations that mimic human abdominal aortic aneurysmal enlargement due to localized losses in elastin [35], we found for the murine thoracic aorta that localized losses of elastic fiber integrity give rise to local dilatations under normotensive conditions, with the initial extent of the insult important in dictating the degree of dilatation over the period studied. Moreover, superimposed hypertension (i.e., uniform increase in pressure) always exacerbated the dilatation, consistent with findings in patients [72], whereas superimposed aging (i.e., additional uniform loss of elastin) had differing effects depending on the degree of the initial insult, which appears to be consistent with findings in Marfan patients whereby stiffness differs from age-matched controls in younger but not older Marfan patients [73].

Natural turnover rates of collagen are orders of magnitude higher than those of elastin [26,64], thus collagen remodeling is a ubiquitous part of most aortic adaptations and maladaptations, including TAAs. Importantly, mutations to the gene that encodes lysyl oxidase (*LOX*), which governs enzymatic cross-linking of collagen, result in aneurysms in humans and mice [74–76]. Note that the normal accumulation of mature collagen is prevented in these germline mutations. Allied studies show further that globally blocking lysyl oxidase in mature mice, in which the aortic collagen had matured, results in a modest phenotype unless superimposed on other defects, particularly Marfan syndrome [77] or a model of aortic aneurysm initiated by the degradation of elastin via elastase [78]. Our results reflect but extend these findings. Whereas use of β-aminopropionitrile (BAPN) in animal models results in uniform reductions in collagen cross-linking, we were able to examine localized reductions. Mild losses had little effect, but severe localized reductions in collagen cross-linking allowed marked dilatations of the thoracic aorta. Interestingly, superimposing hypertension on the vulnerable aorta increased the localized dilatation more than did superimposing aging, though both exacerbated the situation. Localized collagen degradation had similar effects.

Smooth muscle cell-induced vasoconstriction also enables the aortic wall to resist distension under the action of blood pressure. Multiple mutations predisposing to TAAs, including those encoding fibrillin-1 and fibulin-4 and especially those encoding smooth muscle α-actin and smooth muscle myosin heavy chain, compromise vessel-level contractility [16,52,79,80]. Some have thus implied that loss of vessel-level contractility predisposes to TAAs, yet smooth muscle contractility is also compromised in cases wherein TAAs do not develop, including fibulin 5 null mice [81]. We studied potential effects of localized losses in overall contractility, which resulted in modest localized dilatations in the case of an otherwise normal aortic wall exposed to normal pressure loading. Both hypertension and aging increased these local dilatations, though the changes did not reach aneurysmal levels even when blocking contractility fully. Importantly, albeit not shown, marked dilatations arose when both risk factors were superimposed on the initial smooth muscle insult. This simulation is consistent with a study that showed that pressure elevation augments aortic dilatation in the *Acta2*^*-/-*^ mouse, which has reduced vessel-level contractility [82]. Nevertheless, despite some suggestions to the contrary, our simulations suggest that localized losses in vessel-level contractility need not be strong initiators of TAAs. Toward this end, we emphasize that cell-level contractility [80] is controlled by the same actomyosin regulators as is vessel-level contractility [52], with cell-level manifestations affecting both cell migration and the mechanosensing and mechanoregulation of matrix.

Importantly, simulations of potential effects of localized losses of mechanosensing or mechanoregulation of matrix revealed a high propensity toward aneurysmal dilatation. In particular, we modeled compromised mechanosensing by altering the percentage of the intramural stress (*δ*≤1, see Methods) that is transduced to influence matrix production. We have suggested that the propensity of TAAs to form in cases of either fibrillin-1 mutations or smooth muscle cell dysfunction, among others, points to the mechanotransduction axis as a potential initiator of these lesions [6]. Recent studies in cells and different genetically modified mice support this hypothesis [80,83–85]. This in silico study is the first, however, to examine mechanosensing in isolation. The simulations suggest that increasingly greater reductions in localized mechanosensing give rise to increasingly larger localized dilatations (Fig 3), and superimposing aging and especially hypertension increase these dilatations. Because compromised mechanosensing can result from dysfunctional actomyosin activity, comparing these results (fourth rows in Figs 1 and 2) to those for lost vessel-level contractility (third rows in Figs 1 and 2) suggest that compromised mechanosensing has a more severe effect on the aneurysmal phenotype. Whereas vessel-level contractility affects aortic diameter, and thus wall stress [58], mechanosensing affects the ability of the cells to sense the stress and respond appropriately via matrix turnover [6,61]. Compromised mechanoregulation of the newly deposited collagen, modeled via altered deposition stretches, similarly resulted in marked localized dilatations.

Hence, although actomyosin pathways necessarily overlap for both vessel-level contractility and mechanosensing / mechanoregulation of the matrix, the present simulations suggest that aortic consequences are very different. Our findings are thus consistent with an earlier suggestion that the primary role of actomyosin activity in the aorta is “in managing matricellular interactions and sensing the extracellular environment to control appropriate elaboration and maintenance of extracellular matrix” [3]. It is similarly consistent with a prior computational finding that showed that uniform losses in smooth muscle contractility tend to disrupt arterial mechano-adaptations less than does uniformly compromised mechanobiologically mediated matrix turnover [24]. Importantly, our results are also consistent with recent findings that decreased actomyosin activity in smooth muscle cells from *Acta2*^*-/-*^ mice have reduced integrin expression and reduced mechanosensing [86]. In summary, the present simulations further support the hypothesis that dysfunctional mechanosensing is a contributor to TAAs [5–8].

Whereas the present model allows many effects to be studied in isolation, data obtained from actual vessels necessarily reveal intrinsic couplings. To name a few, loss of elastic fiber integrity in a mouse model of Marfan syndrome associates with reduced vasoconstrictive capability [52]; loss of elastic fiber integrity also alters the micro-properties [53] and undulation [87] of the neighboring collagen fibers; deletion of integrin linked kinase alters smooth muscle contractile protein expression, not just cell-matrix interactions [88]; and disruption of fibulin-4, an elastin associated glycoprotein, leads to changes in collagen synthesis and maturation [54] as well as cytoskeletal actin [83,89]. For the purposes of this study, however, we exploited the advantage of in silico simulations and studied in isolation tissue-level consequences of five key genetically predisposed alterations with superimposed hypertension and aging. These simulations support a “multi-hit” hypothesis in that the aorta tends to be mechano-adaptive, and thus fault tolerant, when subjected to mild insults, but it succumbs to severe and especially multiple insults, or hits. Controlling risk factors such as hypertension is critical [90], though some interpretations may suggest otherwise depending on the mouse model used [91].

Notwithstanding the insights gained, there is a need to consider further determinants as well. Endothelial dysfunction can result in reduced nitric oxide bioavailability, hence promoting smooth muscle contractility but also enabling inflammation, which appears to increase with prior cell and matrix damage [92]. Phenotypic modulation of smooth muscle cells from contractile to synthetic can reduce contractility, but also disturb balances in matrix production and removal. Smooth muscle apoptosis necessarily reduces overall vasoconstriction, but also mechanosensing and mechanoregulation of matrix. Losses in elastic fiber integrity reduces wall resilience, but in some cases also the ability of the smooth muscle cells to mechanosense the mechanical state of the wall. There is, therefore, a need to consider many additional combinations of hits, not just superimposed hypertension and aging. We also did not account for initial aortic curvature, which is particularly important in the ascending aorta, or the pulsatility of loading, which includes cyclic distension and extension in the ascending aorta. We did not account for changes in hemodynamics as the aorta enlarges (except indirectly in Figs D, E, F in S1 Supporting Information), though we assumed that wall shear stress was sensed only within the regions that were free of the prescribed insults. Wall shear stress regulation, along with the relative rates of turnover of smooth muscle versus collagen, is critical for maintaining homeostatic control of the distal and proximal segments. Fluid-solid-growth models [93] will enable broader follow-up studies. Finally, we did not account for different ages at which the insult occurred, the sex of the mice, or observed mouse-to-mouse variability in geometry, composition, or wall properties, which would provide better bounds on expected behaviors, particularly why certain lesions appear earlier or progress more rapidly. Clearly more data will be needed to inform more complete models.

Rather, we established a baseline model using mean data from one particular group of male mice, and insults of interest were introduced consistently in an otherwise initially straight circular segment of a model thoracic aorta, which thereby enabled consistent comparisons across groups and clearer delineation of the effects of the insults in isolation and in combination. Not surprisingly, loss of matrix integrity, via either compromised elastic fibers or poorly remodeled collagen, enables marked dilatations. Perhaps surprisingly, compromised mechanosensing and mechanoregulation can evoke as strong of an aneurysmal propensity whereas loss of vessel-level smooth muscle contractility does not. Hence, it appears that reduced actomyosin activity is more severe in its effects in compromising mechanosensing and mechanoregulation of matrix (cf. [60,86,94]). There is a need for caution, therefore, not to over-interpret consequences of compromised vessel-level contractility, often measured via wire or pressure myography [52,79], even though many if not most mutations predisposing to TAAs (e.g., *Fbn1*, *Acta2*, *Myh11*, *Mylk*, *Tgfbr1*, and *Prkg1* mutations in mice) reduce vessel-level contractility.

Whereas we focused on aortic dimensions and properties appropriate for mice, because myriad mouse models are available to study the natural history of TAAs, there will be a need to repeat these simulations for human lesions and it is possible that contractility plays an even lesser role in the human than murine aorta. We otherwise expect, however, that the basic theoretical framework, computational approach, and functional forms of most constitutive equations should translate from the mouse to the human, thus necessitating primarily a re-parameterization of the model when studying human lesions. Consistent with an earlier suggestion that “Over time, the major challenge in aortic aneurysmal research will likely shift from gene identification to the assessment of gene product function in large vessel homeostasis” [3], we submit that in silico studies (including digital twins) can complement in vivo and ex vivo studies as we seek to understand and eventually better treat these complex, multi-factorial lesions, with data-informed patient-specific computations (e.g., [95]) eventually the way forward.

## Supporting information

S1 Appendix Tangent moduli contribution for reoriented / remodeled diagonal fibers via Eq (14). (PDF)

S1 Supporting Information Supplemental Figs A to K. (PDF)

## References

[1] Humphrey JD, Milewicz DM. Aging, smooth muscle vitality, and aortic integrity. Circ Res. 2017, 120: 1849–1851. 10.1161/CIRCRESAHA.117.311075 28596165 PMC5508728

[2] Milewicz DM, Ramirez F. Therapies for thoracic aortic aneurysms and acute aortic dissections: Old controversies and new opportunities. Arterioscler Thromb Vasc Biol. 2019, 39: 126–136. 10.1161/ATVBAHA.118.310956 30651002 PMC6398943

[3] Lindsay ME, Dietz HC. The genetic basis of aortic aneurysm. Cold Spring Harb Perspect. 2014, Med 4: a015909. 10.1101/cshperspect.a015909 PMC4143103 25183854

[4] Brownstein AJ, Ziganshin BA, Kuivaniemi H, Body SC, Bale AE, Elefteriades JA. Genes associated with thoracic aortic aneurysm and dissection. Aorta. 2017, 5: 11–20. 10.12945/j.aorta.2017.17.003 28868310 PMC5570562

[5] Pinard A, Jones GT, Milewicz DM. Genetics of thoracic and abdominal aortic diseases: Aneurysms, dissections, and ruptures. Circ Res. 2019, 124: 588–606. 10.1161/CIRCRESAHA.118.312436 30763214 PMC6428422

[6] Humphrey JD, Tellides G, Schwartz MA, Milewicz DM. Role of mechanotransduction in vascular biology: Focus on thoracic aortic aneurysms and dissections. Circ Res. 2015, 116: 1448–1461. 10.1161/CIRCRESAHA.114.304936 25858068 PMC4420625

[7] Ramirez F, Caescu C, Wondimu E, Galatioto J. Marfan syndrome; A connective tissue disease at the crossroads of mechanotransduction, TGFβ signaling and cell stemness. Matrix Biol. 2018, 71: 82–89. 10.1016/j.matbio.2017.07.004 28782645 PMC5797509

[8] Yamashiro Y, Yanagisawa H. Crossing bridges between extra- and intra-cellular events in thoracic aortic aneurysms. J Atheroscler Thromb. 2018, 25: 99–110. 10.5551/jat.RV17015 28943527 PMC5827090

[9] Cook JR, Clayton NP, Carta L, Galatioto J, Chiu E, Smaldone S, et al Dimorphic effects of transforming growth factor-β signaling during aortic aneurysm progression in mice suggest a combinatorial therapy for Marfan syndrome. Arterioscler Thromb Vasc Biol. 2015, 35: 911–917. 10.1161/ATVBAHA.114.305150 25614286 PMC4376614

[10] Lima BL, Santos EJ, Fernandes GR, Merkel C, Mello MR, Gomes JP, et al A new mouse model for Marfan syndrome presents phenotypic variability associated with the genetic background and overall levels of Fbn1 expression. PLoS One. 2010, 5: e14136 10.1371/journal.pone.0014136 21152435 PMC2994728

[11] Guo DC, Regalado ES, Pinard A, Chen J, Lee K, Rigelsky C, et al LTBP3 pathogenic variants predispose individuals to thoracic aortic aneurysms and dissections. Am J Hum Genet. 2018, 102: 706–712. 10.1016/j.ajhg.2018.03.002 29625025 PMC5985335

[12] Korneva A, Zilberberg L, Rifkin DB, Humphrey JD, Bellini C. Absence of LTBP-3 attenuates the aneurysmal phenotype but not spinal effects on the aorta in Marfan syndrome. Biomech Model Mechanobiol. 2019, 18: 261–273. 10.1007/s10237-018-1080-1 30306291 PMC6367053

[13] Bhushan R, Altinbas L, Jäger M, Zaradzki M, Lehmann D, Timmermann B, et al An integrative systems approach identifies novel candidates in Marfan syndrome-related pathophysiology. J Cell Mol Med. 2019, 23: 2526–2535. 10.1111/jcmm.14137 30677223 PMC6433740

[14] Hansen J, Galatioto J, Caescu CI, Arnaud P, Calizo RC, Spronck B, et al Systems pharmacology-based integration of human and mouse data for drug repurposing to treat thoracic aneurysms. JCI Insight. 2019, 4: e127652. 10.1172/jci.insight.127652 PMC6629138 31167969

[15] Granata A, Serrano F, Bernard WG, McNamara M, Low L, Sastry P, Sinha S. An iPSC-derived vascular model of Marfan syndrome identifies key mediators of smooth muscle cell death. Nat Genet. 2017, 49: 97–109. 10.1038/ng.3723 27893734

[16] Milewicz DM, Prakash SK, Ramirez F. Therapeutics targeting drivers of thoracic aortic aneurysms and acute aortic dissections: insights from predisposing genes and mouse models. Annu Rev Med. 2017, 68: 51–67. 10.1146/annurev-med-100415-022956 28099082 PMC5499376

[17] Milewicz DM, Guo DC, Tran-Fadulu V, Lafont AL, Papke CL, Inamoto S, et al Genetic basis of thoracic aortic aneurysms and dissections: focus on smooth muscle cell contractile dysfunction. Annu Rev Genomics Hum Genet. 2008, 9: 283–302. 10.1146/annurev.genom.8.080706.092303 18544034

[18] El-Hamamsy I, Yacoub MH. Cellular and molecular mechanisms of thoracic aortic aneurysms. Nat Rev Cardiol. 2009, 6: 771–786. 10.1038/nrcardio.2009.191 19884902

[19] Elefteriades JA, Farkas EA. Thoracic aortic aneurysm: clinically pertinent controversies and uncertainties. J Am Coll Cardiol. 2010, 55: 841–857. 10.1016/j.jacc.2009.08.084 20185035

[20] Wilson JS, Baek S, Humphrey JD. Parametric study of effects of collagen turnover on the natural history of abdominal aortic aneurysms. Proc Math Phys Eng Sci. 2013, 469: 20120556 10.1098/rspa.2012.0556 23633905 PMC3637002

[21] Liang L, Liu M, Martin C, Elefteriades JA, Sun W. A machine learning approach to investigate the relationship between shape features and numerically predicted risk of ascending aortic aneurysm. Biomech Model Mechanobiol. 2017, 16: 1519–1533. 10.1007/s10237-017-0903-9 28386685 PMC5630492

[22] Humphrey JD Cardiovascular Solid Mechanics: Cells, Tissues, and Organs. Springer Science & Business Media 2002.

[23] Dajnowiec D, Langille BL. Arterial adaptations to chronic changes in haemodynamic function: coupling vasomotor tone to structural remodelling. Clin Sci. 2007, 113: 15–23. 10.1042/CS20060337 17536999

[24] Valentin A, Humphrey JD. Evaluation of fundamental hypotheses underlying constrained mixture models of arterial growth and remodelling. Philos Trans A Math Phys Eng Sci. 2009, 367: 3585–3606. 10.1098/rsta.2009.0113 19657012 PMC2865879

[25] Latorre M, Humphrey JD. Modeling mechano-driven and immuno-mediated aortic maladaptation in hypertension. Biomech Model Mechanobiol. 2018, 17: 1497–1511. 10.1007/s10237-018-1041-8 29881909 PMC6286240

[26] Humphrey JD. Vascular adaptation and mechanical homeostasis at tissue, cellular, and sub-cellular levels. Cell Biochem Biophys. 2008, 50: 53–78. 10.1007/s12013-007-9002-3 18209957

[27] Latorre M, Humphrey JD. A mechanobiologically equilibrated constrained mixture model for growth and remodeling of soft tissues. Z Angew Math Mech. 2018, 98: 2048–2071. 10.1002/zamm.201700302 30618468 PMC6319907

[28] Cardamone L, Valentin A, Eberth JF, Humphrey JD. Modelling carotid artery adaptations to dynamic alterations in pressure and flow over the cardiac cycle. Math Med Biol. 2010, 27:343–371. 10.1093/imammb/dqq001 20484365 PMC3031348

[29] Galis ZS, Khatri JJ. Matrix metalloproteinases in vascular remodeling and atherogenesis: the good, the bad, and the ugly. Circ Res. 2002, 90:251–262. 11861412

[30] Ruberti JW, Hallab NJ. Strain-controlled enzymatic cleavage of collagen in loaded matrix. Biochem Biophys Res Commun. 2015, 336:483–489. 10.1016/j.bbrc.2005.08.128 16140272

[31] Baek S, Valentín A, Humphrey JD. Biochemomechanics of cerebral vasospasm and its resolution: II. constitutive relations and model simulations. Ann Biomed Eng. 2007, 35(9):1498–1509. 10.1007/s10439-007-9322-x 17487585

[32] Bellini C, Korneva A, Zilberberg L, Ramirez F, Rifkin DB, Humphrey JD. Differential ascending and descending aortic mechanics parallel aneurysmal propensity in a mouse model of Marfan syndrome. J Biomech. 2016, 49: 2383–2389. 10.1016/j.jbiomech.2015.11.059 26755343 PMC4917480

[33] Latorre M, Humphrey JD. Mechanobiological stability of biological soft tissues. J Mech Phys Solid. 2019, 125: 298–325. 10.1016/j.jmps.2018.12.013 PMC6754118 31543547

[34] Baek S, Rajagopal KR, Humphrey JD. A theoretical model of enlarging intracranial fusiform aneurysms. J Biomech Eng. 2006, 128: 142–149. 10.1115/1.2132374 16532628

[35] Wilson JS, Baek S, Humphrey JD. Importance of initial aortic properties on the evolving regional anisotropy, stiffness, and wall thickness of human abdominal aortic aneurysms. J R Soc Interface. 2012, 9: 2047–2058. 10.1098/rsif.2012.0097 22491975 PMC3405751

[36] Latorre M, Humphrey JD. Fast, rate-independent, finite element implementation of a 3D constrained mixture model of soft tissue growth and remodeling. Comput Methods Appl Mech Eng. 2020, 368: 113156 10.1016/j.cma.2020.113156 32655195 PMC7351114

[37] Latorre M, Humphrey JD. Critical roles of time-scales in soft tissue growth and remodeling. APL Bioeng. 2018, 2: 026108 10.1063/1.5017842 31069305 PMC6324203

[38] Latorre M. Modeling biological growth and remodeling: contrasting methods, contrasting needs. Curr Opin Biomed Eng. 2020, 15: 26–31.

[39] Rodriguez EK, Hoger A, McCulloch AD. Stress-dependent finite growth in soft elastic tissues. J Biomech. 1994, 27: 455–467. 10.1016/0021-9290(94)90021-3 8188726

[40] Latorre M, Bersi MR, Humphrey JD. Computational modeling predicts immuno-mechanical mechanisms of maladaptive aortic remodeling in hypertension. Int J Eng Sci. 2019, 141: 35–46. 10.1016/j.ijengsci.2019.05.014 32831391 PMC7437922

[41] Ferruzzi J, Bersi MR, Uman S, Yanagisawa H, Humphrey JD. Decreased elastic energy storage, not increased material stiffness, characterizes central artery dysfunction in fibulin-5 deficiency independent of sex. J Biomech Eng. 2015, 137: 031007. 10.1115/1.4029431 PMC4321117 25532020

[42] Humphrey JD, Tellides G. Central artery stiffness and thoracic aortopathy. Am J Physiol Heart Circ Physiol. 2019, 316: H169–H182. 10.1152/ajpheart.00205.2018 30412443 PMC6880196

[43] Bersi MR, Bellini C, Humphrey JD, Avril S. Local variations in material and structural properties characterize murine thoracic aortic aneurysm mechanics. Biomech Model Mechanobiol. 2019, 18: 203–218. 10.1007/s10237-018-1077-9 30251206 PMC6367054

[44] Laurent S, Boutouyrie P. The structural factor of hypertension: large and small artery alterations. Circ Res. 2015, 116: 1007–1021. 10.1161/CIRCRESAHA.116.303596 25767286

[45] Humphrey JD, Harrison DG, Figueroa CA, Lacolley P, Laurent S. Central artery stiffness in hypertension and aging: A problem with cause and consequence. Circ Res. 2016, 118: 379–381. 10.1161/CIRCRESAHA.115.307722 26846637 PMC4745997

[46] Bersi MR, Bellini C, Wu J, Montaniel KR, Harrison DG, Humphrey JD. Excessive adventitial remodeling leads to early aortic maladaptation in angiotensin-induced hypertension. Hypertension. 2016, 67: 890–896. 10.1161/HYPERTENSIONAHA.115.06262 27001298 PMC4833633

[47] Lakatta EG, Levy D. Arterial and cardiac aging: major shareholders in cardiovascular disease enterprises: Part I: aging arteries: a “set up” for vascular disease. Circulation. 2003, 107: 139–146. 10.1161/01.cir.0000048892.83521.58 12515756

[48] Roccabianca S, Figueroa CA, Tellides G, Humphrey JD. Quantification of regional differences in aortic stiffness in the aging human. J Mech Behav Biomed Mater. 2014, 29: 618–634. 10.1016/j.jmbbm.2013.01.026 23499251 PMC3842391

[49] Jadidi M, Habibnezhad M, Anttila E, Maleckis K, Desyatova A, MacTaggart J, Kamenskiy A. Mechanical and structural changes in human thoracic aortas with age. Acta Biomater. 2020, 103: 172–188. 10.1016/j.actbio.2019.12.024 31877371 PMC6982607

[50] Ferruzzi J, Madziva D, Caulk AW, Tellides G, Humphrey JD. Compromised mechanical homeostasis in arterial aging and associated cardiovascular consequences. Biomech Model Mechanobiol. 2018, 17: 1281–1295. 10.1007/s10237-018-1026-7 29754316 PMC8344131

[51] Dietz HC, Ramirez F, Sakai LY. Marfan’s syndrome and other microfibrillar diseases In: Harris H, Hirschhorn K (eds) Adv Hum Genet. 1994, 22: 153–86. Springer, Boston, MA 10.1007/978-1-4757-9062-7_4 7762452

[52] Chung AW, Au Yeung K, Sandor GG, Judge DP, Dietz HC, Van Breemen C. Loss of elastic fiber integrity and reduction of vascular smooth muscle contraction resulting from the upregulated activities of matrix metalloproteinase-2 and-9 in the thoracic aortic aneurysm in Marfan syndrome. Circ Res. 2007, 101: 512–522. 10.1161/CIRCRESAHA.107.157776 17641224

[53] Lindeman JH, Ashcroft BA, Beenakker JW, van Es M, Koekkoek NB, Prins FA, et al Distinct defects in collagen microarchitecture underlie vessel-wall failure in advanced abdominal aneurysms and aneurysms in Marfan syndrome. Proc Natl Acad Sci. 2010, 107: 862–865. 10.1073/pnas.0910312107 20080766 PMC2818895

[54] Papke CL, Tsunezumi J, Ringuette LJ, Nagaoka H, Terajima M, Yamashiro Y, et al Loss of fibulin-4 disrupts collagen synthesis and maturation: implications for pathology resulting from EFEMP2 mutations. Hum Mol Genet. 2015, 24: 5867–5879. 10.1093/hmg/ddv308 26220971 PMC4581610

[55] Jones JA, Ruddy JM, Bouges S, Zavadzkas JA, Brinsa TA, Stroud RE, et al Alterations in membrane type-1 matrix metalloproteinase abundance after the induction of thoracic aortic aneurysm in a murine model. Am J Physiol Heart Circ Physiol. 2010, 299: H114–H124. 10.1152/ajpheart.00028.2010 20418476 PMC2904124

[56] Davies PF. Flow-mediated endothelial mechanotransduction. Physiol Rev. 1995, 75: 519–560. 10.1152/physrev.1995.75.3.519 7624393 PMC3053532

[57] Li YSJ, Haga JH, Chien S. Molecular basis of the effects of shear stress on vascular endothelial cells. J Biomech. 2005, 38: 1949–1971. 10.1016/j.jbiomech.2004.09.030 16084198

[58] Ferruzzi J, Murtada S-I, Li G, Jiao Y, Uman S, Ting MY, et al Pharmacologically improved contractility protects against aortic dissection in mice with disrupted transforming growth factor-β signaling despite compromised extracellular matrix properties. Arterioscler Thromb Vasc Biol. 2016, 36: 919–927. 10.1161/ATVBAHA.116.307436 26988590 PMC4850095

[59] Vogel V, Sheetz M. Local force and geometry sensing regulate cell functions. Nat Rev Mol Cell Biol. 2006, 7: 265–275. 10.1038/nrm1890 16607289

[60] Humphrey JD, Dufresne ER, Schwartz MA. Mechanotransduction and extracellular matrix homeostasis. Nat Rev Mol Cell Biol. 2014, 15: 802–812. 10.1038/nrm3896 25355505 PMC4513363

[61] Tomasek JJ, Gabbiani G, Hinz B, Chaponnier C, Brown RA. Myofibroblasts and mechano-regulation of connective tissue remodeling. Nat Rev Mol Cell. 2002, Biol 3: 349–363. 10.1038/nrm809 11988769

[62] Cyron CJ, Humphrey JD. Vascular homeostasis and the concept of mechanobiological stability. Int J Eng Sci. 2014, 85: 203–223. 10.1016/j.ijengsci.2014.08.003 25308990 PMC4190482

[63] Humphrey JD, Latorre M. Biomechanics and mechanobiology of extracellular matrix remodeling In: Zhang Y (Ed.) Multi-scale Extracellular Matrix Mechanics and Mechanobiology. Studies in Mechanobiology, Tissue Engineering and Biomaterials. 2020, 23: 1–20, Springer, Cham.

[64] Wagenseil JE, Mecham RP. Vascular extracellular matrix and arterial mechanics. Physiol Rev. 2009, 89: 957–989. 10.1152/physrev.00041.2008 19584318 PMC2775470

[65] Saxena T, Ali AO, Saxena M. Pathophysiology of essential hypertension: an update. Expert Rev Cardiovasc Ther. 2018, 16: 879–887. 10.1080/14779072.2018.1540301 30354851

[66] Najjar SS, Scuteri A, Lakatta EG. Arterial aging: is it an immutable cardiovascular risk factor? Hypertension. 2005, 46: 454–462. 10.1161/01.HYP.0000177474.06749.98 16103272

[67] Safar ME. Arterial aging—hemodynamic changes and therapeutic options. Nat Rev Cardiol. 2010, 7: 442–449. 10.1038/nrcardio.2010.96 20657613

[68] Schwill S, Seppelt P, Grünhagen J, Ott CE, Jugold M, Ruhparwar A, et al The fibrillin-1 hypomorphic mgR/mgR murine model of Marfan syndrome shows severe elastolysis in all segments of the aorta. J Vasc Surg. 2013, 57: 1628–1636. 10.1016/j.jvs.2012.10.007 23294503

[69] MacFarlane EG, Parker SJ, Shin JY, Kang BE, Ziegler SG, Creamer TJ, et al Lineage-specific events underlie aortic root aneurysm pathogenesis in Loeys-Dietz syndrome. J Clin Invest. 2019, 129: 659–675. 10.1172/JCI123547 30614814 PMC6355234

[70] Campobasso R, Condemi F, Viallon M, Croisille P, Campisi S, Avril S. Evaluation of peak wall stress in an ascending thoracic aortic aneurysm using FSI simulations: effects of aortic stiffness and peripheral resistance. Cardiovasc Eng Technol. 2018, 9: 707–722. 10.1007/s13239-018-00385-z 30341731

[71] Sawada H, Chen JZ, Wright BC, Sheppard MB, Lu HS, Daugherty A. Heterogeneity of aortic smooth muscle cells: A determinant for regional characteristics of thoracic aortic aneurysms? J Trans Intern Med. 2018, 6: 93–96. 10.2478/jtim-2018-0023 PMC6231305 30425944

[72] Jondeau G, Boutouyrie P, Lacolley P, Laloux B, Dubourg O, Bourdarias JP, Laurent S. Central pulse pressure is a major determinant of ascending aorta dilation in Marfan syndrome. Circulation. 1999, 99: 2677–2681. 10.1161/01.cir.99.20.2677 10338462

[73] De Wit A, Vis K, Jeremy RW. Aortic stiffness in heritable aortopathies: relationship to aneurysm growth rate. Heart Lung Circ. 2013, 22: 3–11. 10.1016/j.hlc.2012.08.049 22981759

[74] Mäki JM, Räsänen J, Tikkanen H, Sormunen R, Mäkikallio K, Kivirikko KI, Soininen R. Inactivation of the lysyl oxidase gene *Lox* leads to aortic aneurysms, cardiovascular dysfunction, and perinatal death in mice. Circulation. 2002, 106: 2503–2509. 10.1161/01.cir.0000038109.84500.1e 12417550

[75] Guo DC, Regalado ES, Gong L, Duan X, Santos-Cortez RL, Arnaud P, et al *LOX* mutations predispose to thoracic aortic aneurysms and dissections. Circ Res. 2016, 118: 928–934. 10.1161/CIRCRESAHA.115.307130 26838787 PMC4839295

[76] Lee VS, Halabi CM, Hoffman EP, Carmichael N, Leshchiner I, Lian CG, et al Loss of function mutation in LOX causes thoracic aortic aneurysm and dissection in humans. Proc Natl Acad Sci. 2016, 113: 8759–8764. 10.1073/pnas.1601442113 27432961 PMC4978273

[77] Busnadiego O, Gorbenko Del Blanco D, González-Santamaría J, Habashi JP, Calderon JF, Sandoval P, et al Elevated expression levels of lysyl oxidases protect against aortic aneurysm progression in Marfan syndrome. J Mol Cell Cardiol. 2015, 85: 48–57. 10.1016/j.yjmcc.2015.05.008 25988230

[78] Lu G, Su G, Davis JP, Schaheen B, Downs E, Roy RJ, et al A novel chronic advanced stage abdominal aortic aneurysm murine model. J Vasc Surg. 2017, 66: 232–242. 10.1016/j.jvs.2016.07.105 28274752 PMC5483384

[79] Huang J, Gao N, Wang S, Milewicz DM, Kamm KE, Stull JT. Genetic approaches to identify pathological limitations in aortic smooth muscle contraction. PloS One. 2018, 13: e0193769 10.1371/journal.pone.0193769 29494672 PMC5833278

[80] Nolasco P, Fernandes CG, Ribeiro-Silva JC, Oliveira PVS, Sacrini M, de Brito IV, et al Impaired vascular smooth muscle cell force-generating capacity and phenotypic deregulation in Marfan Syndrome mice. Biochim Biophys Acta Mol Basis Dis. 2020, 1866: 165587 10.1016/j.bbadis.2019.165587 31678158

[81] Murtada S-I, Ferruzzi J, Yanagisawa H, Humphrey JD. Reduced biaxial contractility in the descending thoracic aorta of fibulin-5 deficient mice. J Biomech Eng. 2016, 138: 051008 10.1115/1.4032938 26963838 PMC4843850

[82] Chen J, Peters A, Papke CL, Villamizar C, Ringuette LJ, Cao J, et al Loss of smooth muscle α-actin leads to NF-κB–dependent increased sensitivity to angiotensin II in smooth muscle cells and aortic enlargement. Circ Res. 2017, 120: 1903–1915. 10.1161/CIRCRESAHA.117.310563 28461455 PMC5518614

[83] Yamashiro Y, Papke CL, Kim J, Ringuette LJ, Zhang QJ, Liu ZP, et al Abnormal mechanosensing and cofilin activation promote the progression of ascending aortic aneurysms in mice. Sci Signal. 2015, 8: ra105. 26486174 10.1126/scisignal.aab3141 PMC5572214

[84] Yamashiro Y, Thang BQ, Shin SJ, Lino CA, Nakamura T, Kim J, et al Role of thrombospondin-1 in mechanotransduction and development of thoracic aortic aneurysm in mouse and humans. Circ Res. 2018, 123: 660–672. 10.1161/CIRCRESAHA.118.313105 30355232 PMC6211815

[85] Tan KL, Haelterman NA, Kwartler CS, Regalado ES, Lee PT, Nagarkar-Jaiswal S, et al Ari-1 regulates myonuclear organization together with parkin and is associated with aortic aneurysms. Dev Cell. 2018, 45: 226–244. 10.1016/j.devcel.2018.03.020 29689197 PMC5920516

[86] Massett MP, Bywaters BC, Gibbs HC, Trzeciakowski JP, Padgham S, Chen J, et al Loss of smooth muscle α-actin effects on mechanosensing and cell-matrix adhesions. Exp Biol Med. 2020, 245(4): 374–384. 10.1177/1535370220903012 PMC7370591 32064918

[87] Ferruzzi J, Collins MJ, Yeh AT, Humphrey JD. Mechanical assessment of elastin integrity in fibrillin-1-deficient carotid arteries: implications for Marfan syndrome. Cardiovas Res. 2011, 92: 287–295. 10.1093/cvr195 PMC3193833 21730037

[88] Shen D, Li J, Lepore JJ, Anderson TJ, Sinha S, Lin AY, et al Aortic aneurysm generation in mice with targeted deletion of integrin-linked kinase in vascular smooth muscle cells. Circ Res. 2011, 109: 616–628. 10.1161/CIRCRESAHA.110.239343 21778429 PMC3351207

[89] Burger J, van Vliet N, van Heijningen P, Kumra H, Kremers GJ, Alves M, et al Fibulin-4 deficiency differentially affects cytoskeleton structure and dynamics as well as TGFβ signaling. Cell Signal. 2019, 58: 65–78. 10.1016/j.cellsig.2019.02.008 30844428

[90] Kanematsu Y, Kanematsu M, Kurihara C, Tsou TL, Nuki Y, Liang EI, et al Pharmacologically induced thoracic and abdominal aortic aneurysms in mice. Hypertension. 2010, 55: 1267–1274. 10.1161/HYPERTENSIONAHA.109.140558 20212272 PMC2859958

[91] Cassis LA, Gupte M, Thayer S, Zhang X, Charnigo R, Howatt DA, et al ANG II infusion promotes abdominal aortic aneurysms independent of increased blood pressure in hypercholesterolemic mice. Am J Physiol Heart Circ Physiol. 2009, 296: H1660–H1665. 10.1152/ajpheart.00028.2009 19252100 PMC2685354

[92] He R, Guo DC, Sun W, Papke CL, Duraisamy S, Estrera AL, et al Characterization of the inflammatory cells in ascending thoracic aortic aneurysms in patients with Marfan syndrome, familial thoracic aortic aneurysms, and sporadic aneurysms. J Thorac Cardiovasc Surg. 2008, 136: 922–929. 10.1016/j.jtcvs.2007.12.063 18954631 PMC2590650

[93] Figueroa CA, Baek S, Taylor CA, Humphrey JD. A computational framework for fluid–solid-growth modeling in cardiovascular simulations. Comput Methods Appl Mech Eng. 2009, 198: 3583–3602. 10.1016/j.cma.2008.09.013 20160923 PMC2770883

[94] Wozniak MA, Chen CS. Mechanotransduction in development: a growing role for contractility. Nat Rev Mol Cell Biol. 2009, 10: 34–43. 10.1038/nrm2592 19197330 PMC2952188

[95] Mousavi SJ, Farzaneh S, Avril S. Patient-specific predictions of aneurysm growth and remodeling in the ascending thoracic aorta using the homogenized constrained mixture model. Biomech Model Mechanobiol. 2019, 18: 1895–1913. 10.1007/s10237-019-01184-8 31201620

