# Supplementary material for: Numerical knockouts–In silico assessment of factors predisposing to thoracic aortic aneurysms
Source: PLoS Comput Biol. 2020 Oct 20;16(10):e1008273. doi: 10.1371/journal.pcbi.1008273 (PMC7598929; doi:10.1371/journal.pcbi.1008273)
Supplement: S1 Appendix — (PDF) [file pcbi.1008273.s001.pdf]

Supporting Information

Numerical knockouts – In silico assessment of factors predisposing to thoracic aortic aneurysms

PLOS Computational Biology, DOI: 10.1371/journal.pcbi.1008273

Marcos Latorre, Jay D. Humphrey

Department of Biomedical Engineering, Yale University, New Haven, CT, USA

S1 Appendix. Tangent moduli contribution for reoriented / remodeled diagonal fibers via Eq. (14)

The second Piola-Kirchhoff stresses for the diagonal collagen fibers reads (cf. Eq. (40) in [36])

$$\mathbf{S}_h^{cd} = J_h \phi_h^{cd} \mathbf{U}_h^{-1} \hat{\boldsymbol{\sigma}}_{\mathrm{N}h}^{cd} \mathbf{U}_h^{-1} = J_h \phi_h^{cd} \hat{\boldsymbol{\sigma}}_{\mathrm{N}h}^{cd} : \mathbf{U}_h^{-1} \odot \mathbf{U}_h^{-1} = J_h \phi_h^{cd} \mathbf{U}_h^{-1} \odot \mathbf{U}_h^{-1} : \hat{\boldsymbol{\sigma}}_{\mathrm{N}h}^{cd}$$

where the rotated Cauchy stress at the constituent level is

$$\hat{\boldsymbol{\sigma}}_{\mathrm{N}h}^{cd} = \hat{\sigma}^c \mathbf{N}_h^d \otimes \mathbf{N}_h^d$$

whose magnitude $\hat{\sigma}^c$ (depending on G^c only) yet remains constant here though not its orientation

$$\mathbf{N}_h^d = \mathbf{N}^d(\alpha_{0h}) = \boldsymbol{e}_\theta \sin\alpha_{0h} \pm \boldsymbol{e}_z \cos\alpha_{0h}$$

with α_{0h} expressed in terms of the right Cauchy-Green tensor $\mathbf{C}$ through Eq. (14)

$$\tan\alpha_{0h} = \frac{\lambda_{\theta h}}{\lambda_{zh}} \tan\alpha_{0o} = \frac{\sqrt{\mathbf{C}: \boldsymbol{e}_\theta \otimes \boldsymbol{e}_\theta}}{\sqrt{\mathbf{C}: \boldsymbol{e}_z \otimes \boldsymbol{e}_z}} \tan\alpha_{0o}.$$

The fourth-order tangent moduli tensor associated with the change in orientation of $\hat{\boldsymbol{\sigma}}_{\mathrm{N}h}^{cd}$ is

$$\mathbb{C}_{\alpha_h} = 2 \left. \frac{\partial \mathbf{S}_h^{cd}}{\partial \mathbf{C}} \right|_{\alpha_{0h}} = J_h \phi_h^{cd} \mathbf{U}_h^{-1} \odot \mathbf{U}_h^{-1} : 2 \frac{\partial \hat{\boldsymbol{\sigma}}_{\mathrm{N}h}^{cd}}{\partial \mathbf{C}}$$

where

$$2 \frac{\partial \hat{\boldsymbol{\sigma}}_{\mathrm{N}h}^{cd}}{\partial \mathbf{C}} = \frac{\partial \hat{\boldsymbol{\sigma}}_{\mathrm{N}h}^{cd}}{\partial \tan\alpha_{0h}} \otimes 2 \frac{\partial \tan\alpha_{0h}}{\partial \mathbf{C}} = \hat{\sigma}^c \left(\frac{\partial \mathbf{N}_h^d}{\partial \tan\alpha_{0h}} \otimes \mathbf{N}_h^d + \mathbf{N}_h^d \otimes \frac{\partial \mathbf{N}_h^d}{\partial \tan\alpha_{0h}} \right) \otimes 2 \frac{\partial \tan\alpha_{0h}}{\partial \mathbf{C}}$$

with

$$\mathbf{N}_{h,\alpha_{0h}}^d := \frac{\partial \mathbf{N}_h^d}{\partial \tan\alpha_{0h}} = \boldsymbol{e}_\theta \frac{d(\sin\alpha_{0h})}{d(\tan\alpha_{0h})} \pm \boldsymbol{e}_z \frac{d(\cos\alpha_{0h})}{d(\tan\alpha_{0h})} = \frac{\boldsymbol{e}_\theta \mp \boldsymbol{e}_z \tan\alpha_{0h}}{(1+\tan^2\alpha_{0h})^{3/2}}$$

and

$$2 \frac{\partial \tan\alpha_{0h}}{\partial \mathbf{C}} = \left(2 \frac{1}{\lambda_{\theta h}} \frac{\partial \lambda_{\theta h}}{\partial \mathbf{C}} - 2 \frac{1}{\lambda_{zh}} \frac{\partial \lambda_{zh}}{\partial \mathbf{C}} \right) \frac{\lambda_{\theta h}}{\lambda_{zh}} \tan\alpha_{0o} = \left(\frac{1}{\lambda_{\theta h}^2} \boldsymbol{e}_\theta \otimes \boldsymbol{e}_\theta - \frac{1}{\lambda_{zh}^2} \boldsymbol{e}_z \otimes \boldsymbol{e}_z \right) \tan\alpha_{0h}.$$

Hence

$$\mathbb{C}_{\alpha_{0h}} = J_h \phi_h^{cd} \hat{\sigma}^c \tan \alpha_{0h} \left(\mathbf{U}_h^{-1} \mathbf{N}_{h,\alpha_{0h}}^d \otimes \mathbf{U}_h^{-1} \mathbf{N}_h^d + \mathbf{U}_h^{-1} \mathbf{N}_h^d \otimes \mathbf{U}_h^{-1} \mathbf{N}_{h,\alpha_{0h}}^d \right) \otimes \left(\frac{\boldsymbol{e}_\theta \otimes \boldsymbol{e}_\theta}{\lambda_{\theta h}^2} - \frac{\boldsymbol{e}_z \otimes \boldsymbol{e}_z}{\lambda_{zh}^2} \right)$$

which is a contribution to be added to Eq. (42) in [36]. If needed in spatial form

$$\mathbb{c}_{\alpha_{0h}} = \phi_h^{cd} \hat{\sigma}^c \tan \alpha_{0h} \left(\mathbf{R}_h \mathbf{N}_{h,\alpha_{0h}}^d \otimes \mathbf{R}_h \mathbf{N}_h^d + \mathbf{R}_h \mathbf{N}_h^d \otimes \mathbf{R}_h \mathbf{N}_{h,\alpha_{0h}}^d \right) \otimes \left(\frac{\mathbf{F}_h \boldsymbol{e}_\theta \otimes \mathbf{F}_h \boldsymbol{e}_\theta}{\lambda_{\theta h}^2} - \frac{\mathbf{F}_h \boldsymbol{e}_z \otimes \mathbf{F}_h \boldsymbol{e}_z}{\lambda_{zh}^2} \right)$$

where $\mathbf{F}_h = \mathbf{R}_h \mathbf{U}_h$.
