# Supplementary material for: Numerical knockouts–In silico assessment of factors predisposing to thoracic aortic aneurysms
Source: PLoS Comput Biol. 2020 Oct 20;16(10):e1008273. doi: 10.1371/journal.pcbi.1008273 (PMC7598929; doi:10.1371/journal.pcbi.1008273)
Supplement: S1 Supporting Information — (PDF) [file pcbi.1008273.s002.pdf]

Supporting Information

Numerical knockouts – In silico assessment of factors predisposing to thoracic aortic aneurysms

PLOS Computational Biology, DOI: 10.1371/journal.pcbi.1008273

Marcos Latorre, Jay D. Humphrey

Department of Biomedical Engineering, Yale University, New Haven, CT, USA

S1 Supporting Information — Supplemental Figures A to K

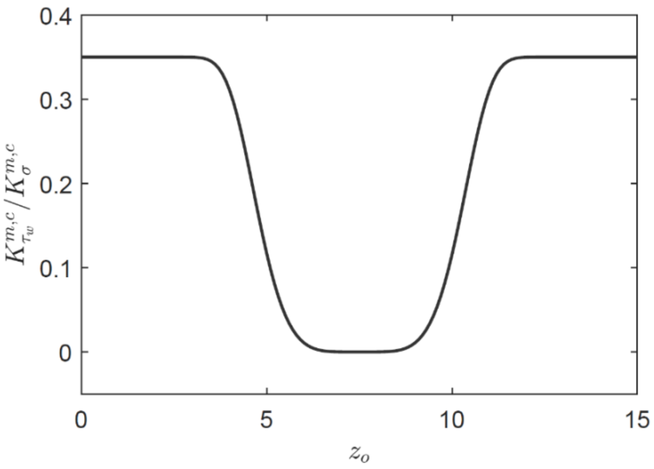

Fig A. Axially nonuniform distribution, Eq. (9), for the ratio of shear-to-intramural stress mediated gains $\mathrm{K}^{m,c}_{\tau_w}/\mathrm{K}^{m,c}_{\sigma}$ (with $\mathrm{K}^{m,c}_{\tau_w}/\mathrm{K}^{m,c}_{\sigma}|_{end} = 0.35$, $\mathrm{K}^{m,c}_{\tau_w}/\mathrm{K}^{m,c}_{\sigma}|_{central} = 0$, $\nu_z = 5$, $z_{od} = 3\mathrm{mm}$) prescribed for the simulated nonuniform dilatations of the aortic wall. That is, we assumed that changes in hemodynamics arising from aortic enlargement result in changing wall shear stresses that are sensed only within the regions that remained free of the prescribed insults and remained cylindrical (with $\tau_{wh}/\tau_{wo} \simeq Q_h a_o^3/(Q_o a_h^3)$ assumed in these regions).

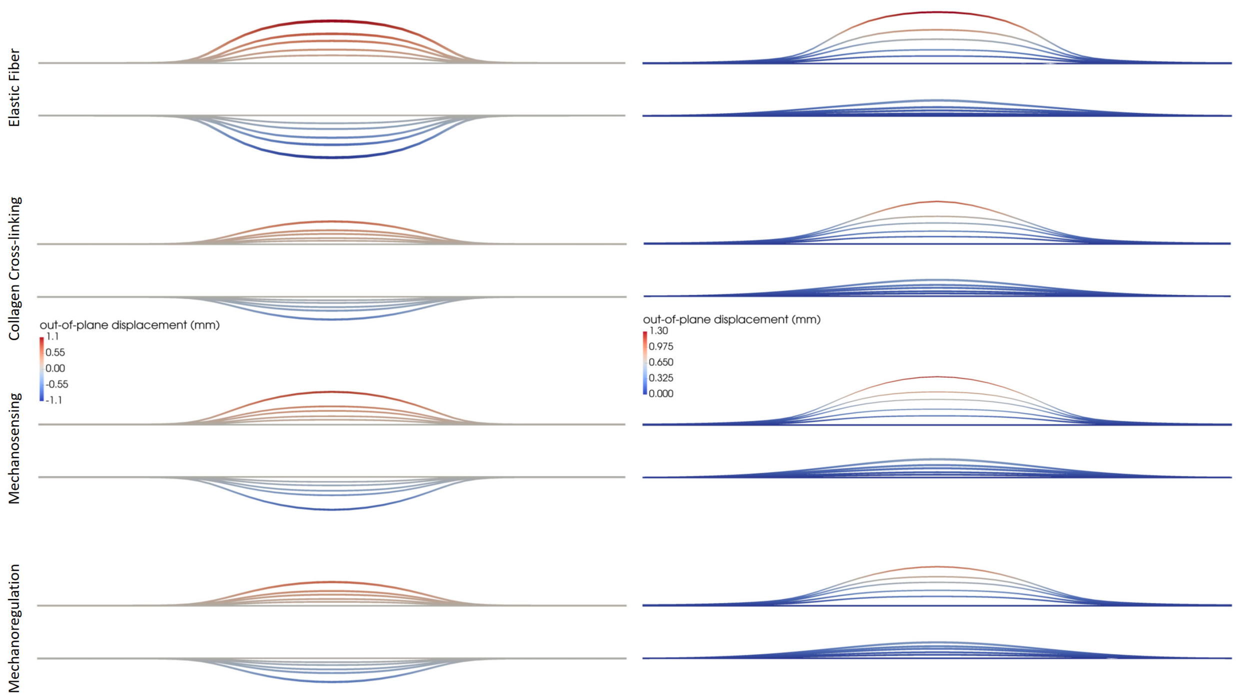

Fig B. Mechanobiologically (quasi)equilibrated, progressive axisymmetric (left) or asymmetric (right) dilatations of an initial non-aneurysmal cylindrical aortic segment with increasingly greater (0%, 40%, 60%, 80%, 90%, and 100% relative to maximum, see Table 1) losses of elastic fiber integrity (first row, parameter c^e), collagen cross-linking (second row, parameter c_1^c), mechanosensing (third row, parameter δ), or mechanoregulation (fourth row, parameter G^c). Note the nonlinearly increasing character of all dilatations, with enlargements developing from 60% to 80% prescribed insults greater than those from 40% to 60%, and similarly for those arising from 90% to 100% insults relative to those from 80% to 90%. Shown are longitudinal sections with superimposed color maps for the transverse displacement in Cartesian coordinates (positive upwards) for the mechanically-loaded geometries, with preserved inner pressure $P_h = P_o$ and fixed axial displacements at the ends. Note the initial luminal radius $a_o = 0.647\text{mm}$, hence maximal changes in diameter at the central cross section of $d_{max}/d_o \approx 2.7$ (axisymmetric) and $d_{max}/d_o \approx 2$ (asymmetric), which are aneurysmal. Such simulations helped to define the individual "mild" and "severe" cases in Figures 1 and 2 in the paper. Specifically, the parameters associated with the "severe phenotype" indicated the most severe case wherein the solution converged using a standard Newton-Raphson solver. It appears that beyond this severe case reflects a local loss of mechanobiological stability [33], though a formal stability analysis was not performed. Similarly, the parameters associated with the "mild phenotype" indicated the most severe case for which superposition of either hypertension or aging resulted in loss of convergence of the solution.

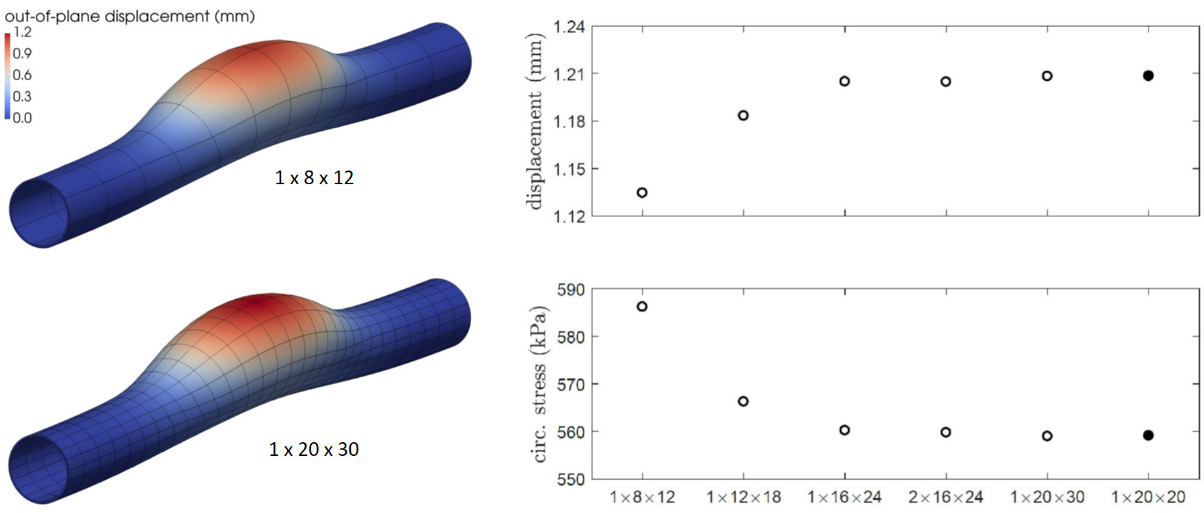

Fig C. Convergence study for quadratic meshes that comprise different numbers of elements in radial (N_r), circumferential (N_θ), and axial (N_z) directions, ranging from $N_r \times N_\theta \times N_z = 1 \times 8 \times 12$ (coarse mesh, top left) to $1 \times 20 \times 30$ (finer mesh, bottom left) elements for an asymmetric aneurysm that originates from localized losses of mechanosensing. Shown are color maps for out-of-plane displacements (colorimetric scale) as well as convergence plots for the out-of-plane displacement (top right) and circumferential Cauchy stress (bottom right) at the apex (mid-thickness). Note that consideration of two quadratic elements through the thickness does not refine further the computed solution for this uni-layered wall model (compare results for meshes $1\times 16 \times 24$ and $2 \times 16 \times 24$), and that the mesh used for all the simulations in the main paper ($1 \times 20 \times 20$ – filled symbol) yields accurate results relative to the finer meshes shown ($2 \times 16 \times 24$ and $1 \times 20 \times 30$). Albeit not shown, radial stresses (e.g., at the apex) similarly converged to prescribed traction (pressure) conditions on the inner ($\sigma_{rrh} \to -P_h = -P_o = -13.98\ \mathrm{kPa}$) and outer ($\sigma_{rrh} \to 0$) boundaries.

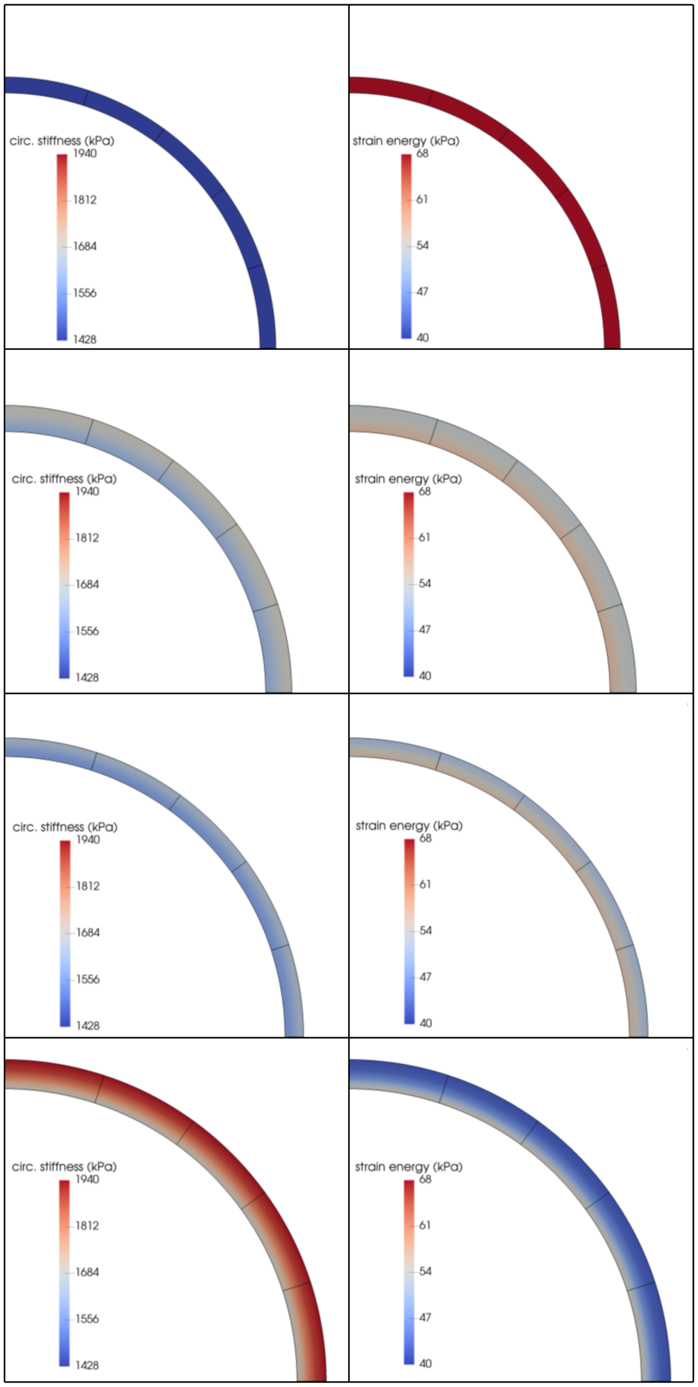

Fig D. Initial (first row) and evolved (other rows) homeostatic states resulting from a *uniform* 1.5-fold elevation of blood pressure (second row, modeling hypertension), a *uniform* 30% loss of elastic fiber integrity (third row, modeling vascular aging), or both (fourth row). Shown are color maps for circumferential material stiffness (left) and elastic energy storage per unit current volume (right) for the mechanically-loaded geometries. Note the original in vivo homeostatic values $c_{\theta\theta\theta\theta o} = 1428\ \text{kPa}$ (dark blue, bottom mark) and $W_o = 68\ \text{kPa}$ (dark red, top mark). These uniform insults resulted in uniform wall thickening or dilatations.

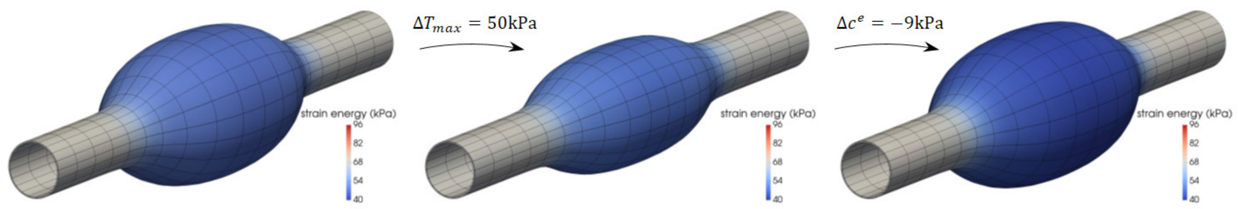

Fig E. Potential protective role of smooth muscle contractility in cases of compromised elastic fiber integrity. Note that partially increased contractility reduces the extent of the dilatation (center; $T_{max} = 50\text{kPa}$) when superimposed on an aneurysm that arose from a prior localized 62% loss of elastic fiber integrity (left; cf. panel "severe" within the first row in Figure 1). This protection is yet overcome by an additional 10% degradation of elastin (right; localized 72% loss of elastic fiber integrity, with $T_{max} = 50\ \text{kPa}$; note a further decrease in elastic energy storage capability), hence revealing complex interactions between two key mechanical contributors to normal wall structure, smooth muscle, and elastin.

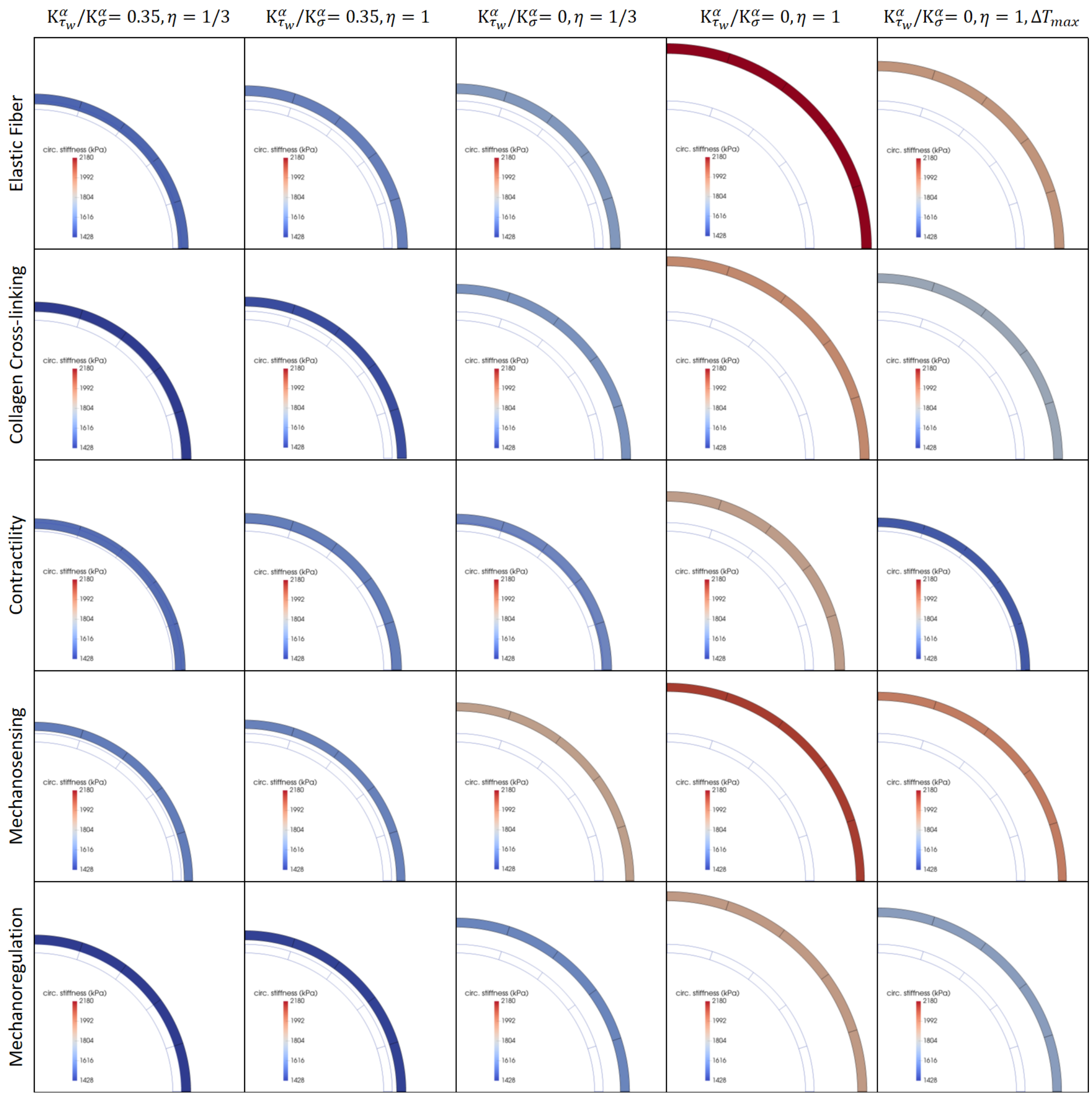

Fig F. Evolved homeostatic states resulting from *uniform* insults: a 30% loss of elastic fiber integrity (first row), 20% decrease in collagen cross-linking (second row), 80% loss of smooth muscle contractility (third row), 12% loss of mechanosensing (fourth row), or 1% reduction in collagen deposition stretch (fifth row), for different sets of G&R parameters: $K^{\alpha}_{\tau_w}/K^{\alpha}_{\sigma} = 0.35$ and $\eta = 1/3$ (first column), $K^{\alpha}_{\tau_w}/K^{\alpha}_{\sigma} = 0.35$ and $\eta = 1$ (second column), $K^{\alpha}_{\tau_w}/K^{\alpha}_{\sigma} = 0$ and $\eta = 1/3$ (third column), $K^{\alpha}_{\tau_w}/K^{\alpha}_{\sigma} = 0$ and $\eta = 1$ (fourth column), with contractile capacity ($\Delta T_{max} = 150$ kPa) added to the right-most (fifth) column. Recall from Methods that $\eta = (k^m/k^c)(K^m_i/K^c_i)$ denotes a ratio of rates of smooth muscle-to-collagen turnover. Colorimetric scales show (elemental) circumferential material stiffness for the mechanically-loaded geometries. Note the original in vivo homeostatic value $c_{\theta\theta\theta\theta o} = 1428$ kPa (dark blue, bottom mark).

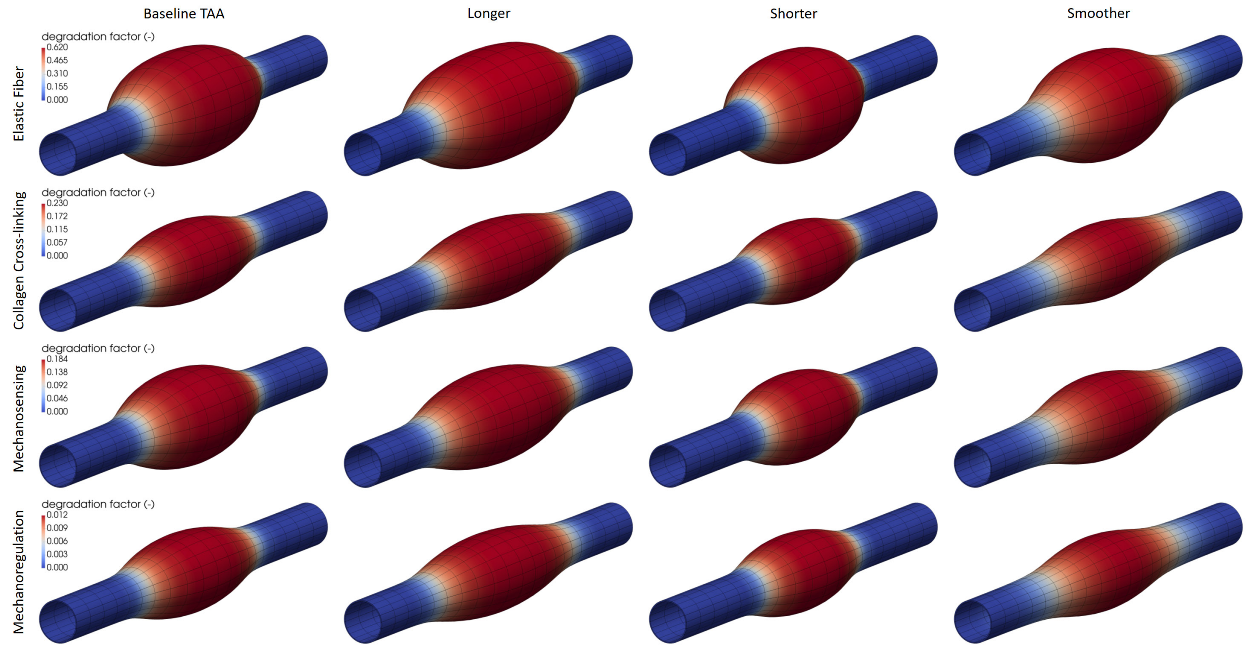

Fig G. Fully developed, mechanobiologically equilibrated, axisymmetric dilatations of an initially cylindrical aortic segment caused by severe localized (via the respective parameter $\vartheta_{central}$ in Eq. (9)) loss of elastic fiber integrity (first row), decrease in collagen cross-linking (second row), compromised mechanosensing (third row), or compromised mechanoregulation (fourth row) with spatially different axial distributions of prescribed damage $\vartheta(z_o)$ (whose relative loss is shown by the color map) via the decay (ν_z) and deviation (z_{od}) parameters in Eq. (9). Results are shown for: $\nu_z = 5$ and $z_{od} = 3\ \mathrm{mm}$ (baseline aneurysms, first column), $\nu_z = 5$ and $z_{od} = 3.75\ \mathrm{mm}$ (longer aneurysms, second column), $\nu_z = 5$ and $z_{od} = 2.5\ \mathrm{mm}$ (shorter aneurysms, third column), and $\nu_z = 3$ and $z_{od} = 3.75\ \mathrm{mm}$ ("longer", but notably reduced, aneurysms afforded by smoother transitions from non-damaged to damaged regions, fourth column).

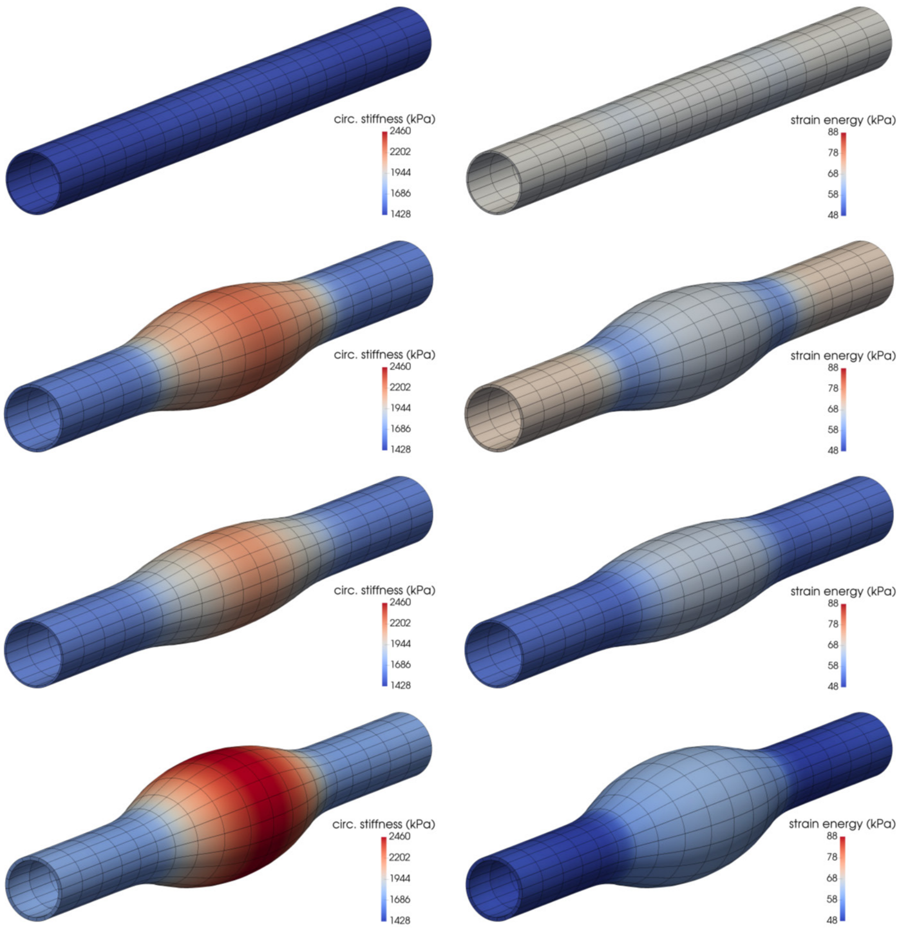

Fig H. Fully developed, mechanobiologically equilibrated, axisymmetric dilatations of an initially cylindrical aortic segment (top panels) caused by mild locally compromised degradations of collagen, via $\eta_{central}$ in Eq. (9), with superimposed risk factors of hypertension (second row, 50% increase in blood pressure, with $\eta = 1.11$), aortic aging (third row, uniform 15% loss of elastic fiber integrity, with $\eta = 1.125$), or both combined (bottom panels, with $\eta = 1.05$). Shown are color maps for circumferential material stiffness (left) and elastic energy storage per unit current volume (right) for the mechanically-loaded geometries. Note the original in vivo homeostatic values $c_{\theta\theta\theta\theta o} = 1428\,\mathrm{kPa}$ (dark blue, bottom mark) and $W_o = 68\,\mathrm{kPa}$ (light grey, centered mark). Both $\mathrm{K}^{\alpha}_{\tau_w}/\mathrm{K}^{\alpha}_{\sigma}|_{end} > 0$ and $\eta_{end} < 1$ were adjusted via Eq. (9) to maintain the distal and proximal segments normal (cf. Figures A and F).

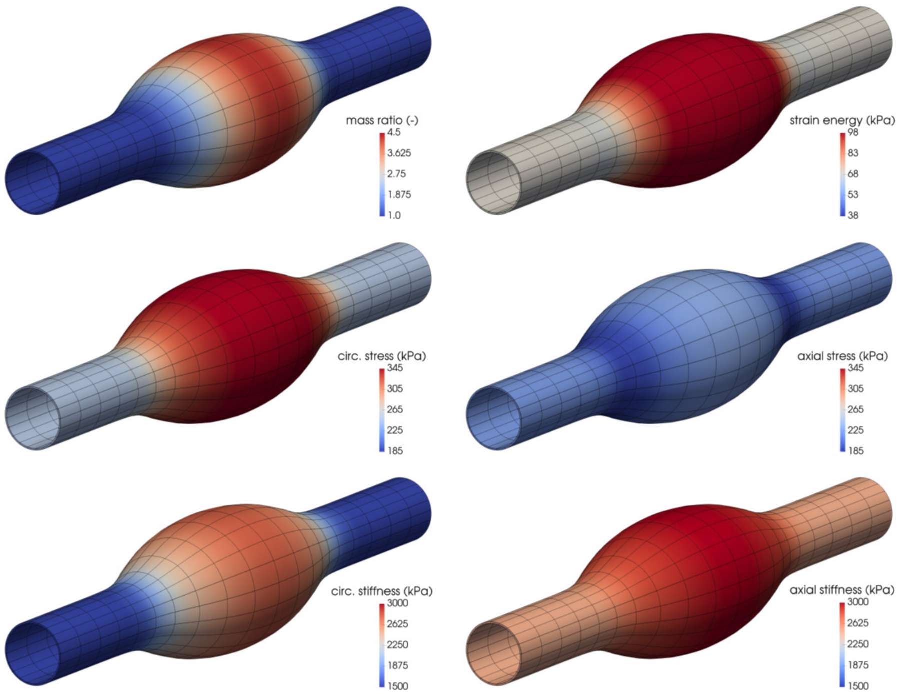

Fig I. Fully developed, mechanobiologically equilibrated, axisymmetric dilatations caused by a marked ($\delta = 0.185$) locally compromised mechanosensing in isolation. Shown are color maps for the Jacobian (equivalently, mass ratio) J_h, elastic energy storage per unit current volume W_h, circumferential $\sigma_{\theta\theta h}$ and axial σ_{zzh} Cauchy stress, and circumferential $c_{\theta\theta\theta\theta h}$ and axial c_{zzzzh} material stiffness. Note the original in vivo homeostatic values $J_o = 1$, $W_o = 68$ kPa, $\sigma_{\theta\theta o} = 225$ kPa, $\sigma_{zzo} = 250$ kPa, $c_{\theta\theta\theta\theta o} = 1428$ kPa, and $c_{zzzzo} = 3275$ kPa.

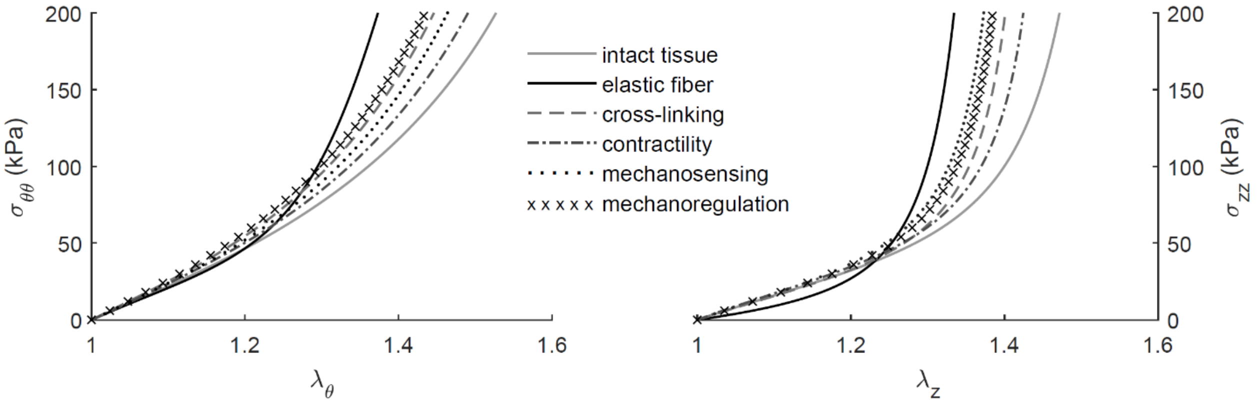

Fig J. Calculated circumferential (left) and axial (right) Cauchy stress-stretch behaviors for simulated equibiaxial stress-testing protocols at the apices of localized lesions resulting from five of the different insults studied: 62% loss of elastic fiber integrity (black solid lines), 23% decrease in collagen cross-linking (dashed lines), 100% loss of smooth muscle contractility (dashed-dotted lines), 18.5% loss of mechanosensing (dotted lines), and 1.2% decrease in collagen deposition stretch (crossed lines). Evolved material properties and mass fractions were extracted in post-processing from the simulated dilatations of the aortic wall (without added effects of hypertension or aging) in Figures 1 or 2, respectively. The simulated initial nonaneurysmal equibiaxial behaviors (grey solid lines) are included for comparison. Note that in vivo in-plane stresses reach $\sim 200\ \mathrm{kPa}$ consistent with measurements for mouse descending thoracic aortas.

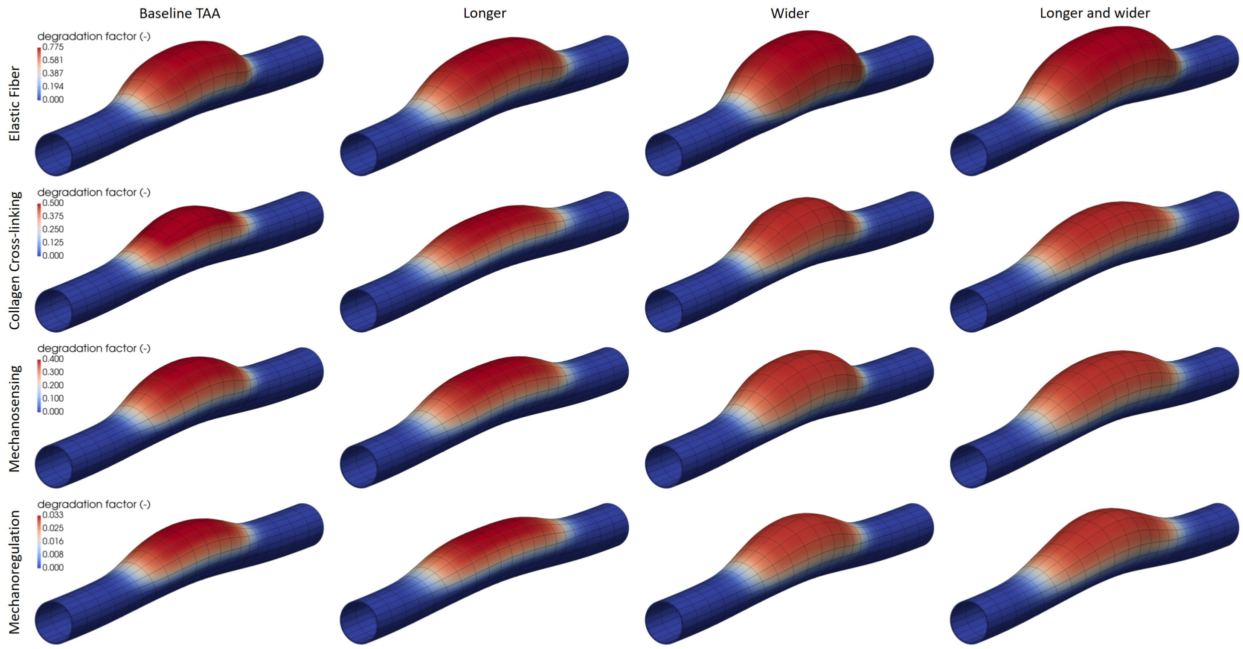

Fig K. Fully developed, mechanobiologically equilibrated, asymmetric dilatations of an initially cylindrical aortic segment caused by a severe localized (via the respective parameter ϑ_{apex} in Eq. (10)) loss of elastic fiber integrity (first row), decrease in collagen cross-linking (second row), compromised mechanosensing (third row), or compromised mechanoregulation (fourth row), with spatially different axial and circumferential distributions of damage $\vartheta(z_o, \theta_o)$ (whose relative loss is shown by the color map) prescribed via the deviation parameters z_{od} and θ_{od} in Eq. (10). Results are shown for: $z_{od} = 3\ \mathrm{mm}$ and $\theta_{od} = \pi/3$ (baseline aneurysms, first column), $z_{od} = 3.75\ \mathrm{mm}$ and $\theta_{od} = \pi/3$ (longer aneurysms, second column), $z_{od} = 3\ \mathrm{mm}$ and $\theta_{od} = \pi/2.5$ (wider aneurysms, third column), and $z_{od} = 3.75\ \mathrm{mm}$ and $\theta_{od} = \pi/2.5$ (longer and wider aneurysms, fourth column). Note that greater involvements of compromised properties along the circumferential direction tend to cause larger dilatations.
